# *PpMYB123*-mediated proanthocyanidin accumulation alleviates bacterial spot disease in peach

**DOI:** 10.1093/hr/uhag032

**Published:** 2026-01-30

**Authors:** Lei Zhao, Di Ai, Zhaoyang Li, Miaoyi Li, Chaoxi Luo, Qian Peng, Yuepeng Han, Jian-Ping An

**Affiliations:** State Key Laboratory of Plant Diversity and Specialty Crops, Wuhan Botanical Garden, Chinese Academy of Sciences, Wuhan 430074, China; University of Chinese Academy of Sciences, 19A Yuquanlu, Beijing 100049, China; University of Chinese Academy of Sciences, 19A Yuquanlu, Beijing 100049, China; University of Chinese Academy of Sciences, 19A Yuquanlu, Beijing 100049, China; Hubei Key Laboratory of Plant Pathology, College of Plant Science and Technology, Huazhong Agricultural University, Wuhan 430070, China; Hubei Key Laboratory of Economic Forest Germplasm Improvement and Resources Comprehensive Utilization, Huanggang Normal University, Huanggang 438000, China; State Key Laboratory of Plant Diversity and Specialty Crops, Wuhan Botanical Garden, Chinese Academy of Sciences, Wuhan 430074, China; State Key Laboratory of Plant Diversity and Specialty Crops, Wuhan Botanical Garden, Chinese Academy of Sciences, Wuhan 430074, China

## Abstract

Bacterial spot (BS) disease significantly impairs vigor, fruit quality, and yield in peach trees. However, research on this disease remains limited. In this study, peach leaves and fruits were inoculated with the pathogen isolated from infected leaves, triggering a robust accumulation of proanthocyanidins (PA) in both tissues. Further investigation revealed that pathogen inoculation promoted PA accumulation by upregulating *PpMYB123*, which transactivated the core PA biosynthetic genes *PpANR* and *PpLAR*. Notably, the E3 ubiquitin ligase PpPUB23 negatively regulated PpMYB123. However, its transcript levels were significantly suppressed following inoculation, thereby stabilizing PpMYB123 and enhancing PA production. PA conferred dual protection by scavenging excess reactive oxygen species (ROS) and suppressing pathogen growth. Our findings provide molecular evidence for PA-mediated defense against BS disease in peach.

## Introduction

Pathogenic infections caused by microbial agents, such as bacteria and fungi, initiate a cascade of physiological disturbances in plants, including reactive oxygen species (ROS) bursts that can lead to cellular damage. This cellular damage impairs essential physiological processes, such as photosynthetic efficiency and biomass accumulation, ultimately reducing crop yield and marketable quality [1–8]. This biotic stress in plants is counteracted by a complex defense system that involves activating resistance (R) genes, producing hormones, such as salicylic acid (SA) and jasmonic acid (JA), and triggering the synthesis of defensive secondary metabolites [9–13].

The phenylpropanoid pathway generates a wide array of specialized metabolites, including lignin, flavonol, anthocyanin, and proanthocyanidins (PAs). These metabolites play vital roles in plant disease resistance. Lignin, a predominant component of secondary cell walls, confers mechanical reinforcement to prevent pathogen penetration and colonization by increasing cell wall rigidity [14–16]. Flavonol, anthocyanin, and PAs possess multiple hydroxyl groups and a special conjugated structure of the benzene ring. This structure enables them to neutralize ROS through electron transfer or metal chelation and reduce oxidative damage to suppress the occurrence of disease [17–21]. PAs in plants exist as polymeric structures with abundant hydroxyl groups, exhibiting strong resistance to degradation. Moreover, PAs collaborate with lignin to reinforce cell walls, thus playing a vital role in disease resistance. The accumulation of PAs has been found to enhance resistance against fungal diseases in poplar [22, 23]. PA enrichment enhances resistance to *Colletotrichum gloeosporioides* and significantly reduces susceptibility to powdery mildew in grapevine [20, 24, 25].

The biosynthesis of PAs in plants is regulated by two key enzymatic steps: anthocyanidin reductase (ANR) and leucoanthocyanidin reductase (LAR). ANR catalyzes the conversion of anthocyanins into epicatechin derivatives, whereas LAR mediates the formation of catechin precursors [26]. This metabolic process is strictly regulated by environmental stresses, such as strong light, cold, drought, and pathogen infection [20, 23, 27–29], as well as hormone signals including SA and JA [30–32]. The PA biosynthesis gene is regulated by the transcription complex formed by MYB transcription factors, bHLH co-activators, and WD40 repeat proteins, with MYB transcription factor playing a key role. AtTT2/AtMYB123 in *Arabidopsis* directly binds to the *AtANR*/*BAN* promoter to drive PA deposition in seed coats [33]. Subsequent studies revealed the conservation of MYB-mediated PA accumulation in various plant species, for example, the involvement of *VvMYBPA1*, *VvMYB5b*, and *VvMYBPA2* in grape [34–36] and *FaMYB9*, *FaMYB11*, and *FaMYB5* in strawberry [37, 38].

Peach (*Prunus persica*) is a commercially vital stone fruit crop, prized for both its ornamental and culinary applications. However, it faces significant threats from bacterial pathogens, compromising its aesthetic value and fruit marketability. Specifically, bacterial spot (BS) disease results in leaf perforations and necrotic fruit lesions; however, its molecular mechanisms remain poorly elucidated. Our study revealed that pathogen inoculation triggered pronounced PA accumulation in peach leaves and fruits through a novel PpPUB23–PpMYB123 regulatory module. This inoculation upregulated the transcription of *PpMYB123*, promoting PA accumulation through the transcriptional activation of *PpANR* and *PpLAR*. The E3 ubiquitin ligase PpPUB23 negatively regulated the function of PpMYB123. However, pathogen inoculation significantly downregulated the expression of *PpPUB23*, leading to increased PA accumulation. Thus, PAs confer dual protective mechanisms against pathogen inoculation by removing excessive ROS and suppressing pathogen growth.

## Results

### PA specifically accumulates under pathogenic bacteria inoculation in peach

**Figure 1:**
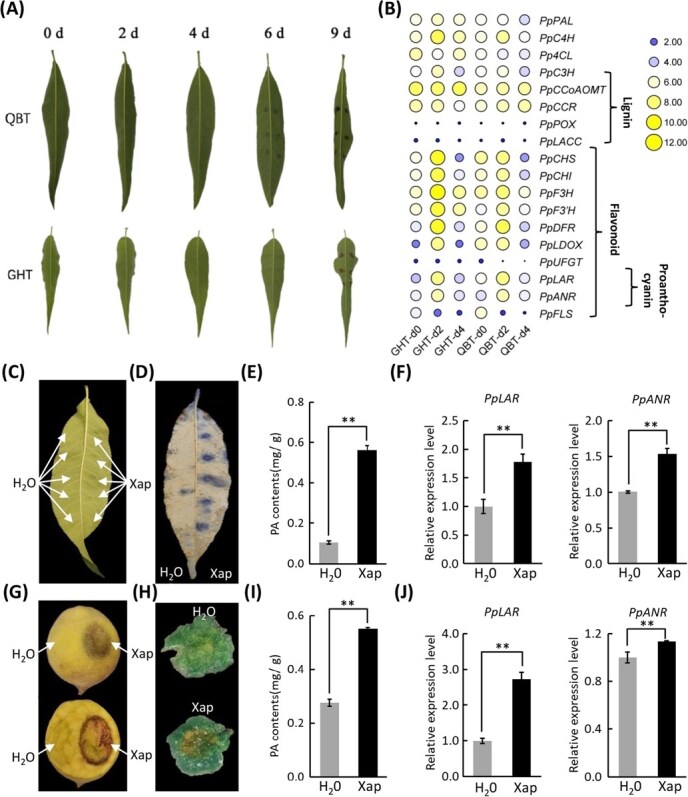
PA specifically accumulates in response to pathogenic bacterial inoculation in peach. (A) Pathogen inoculation induces phenotypic changes in the leaves of ‘QBT’ and ‘GHT’. (B) Pathogen inoculation alters the expression profiles of genes involved in the phenylpropanoid biosynthesis pathway. (C) Pathogen inoculation induces phenotypic changes in the leaves of ‘XHJ’. (D) DMACA staining of pathogen-inoculated leaves. (E) Quantification of PA content in pathogen-inoculated leaf tissues. (F) Expression profiles of *PpLAR* and *PpANR* in pathogen-inoculated leaf tissues. (G) Pathogen inoculation induces phenotypic changes in the fruits of ‘XHJ’. (H) DMACA staining of pathogen-inoculated fruits tissues. (I) Quantification of PA content in pathogen-inoculated fruit tissues. (J) Expression profiles of *PpLAR* and *PpANR* in pathogen-inoculated fruit tissues. Error bars in (E), (F), (I), and (J) represent the standard error (SE) of three biological replicates.

To investigate the pathogenesis of BS disease in peach, healthy leaves from ‘QBT’ and ‘GHT’ were selected for pathogen inoculation. Phenotypic characterization showed initial necrotic lesions at inoculation sites in both genotypes by 4 days post-inoculation (dpi), with lesion expansion culminating in cell death by 9 dpi (Fig. 1A). To elucidate the transcriptional regulatory networks involved in the disease response, RNA-seq-based transcriptome profiling was performed on leaf tissues collected at 0 (control), 2, and 4 dpi. Differential expression analysis (|log2FC| ≥ 1, FDR < 0.05) identified 1097 commonly upregulated and 1961 commonly downregulated genes at 2 dpi in both genotypes. At 4 dpi, 1185 genes were upregulated and 1723 downregulated. Intersection analysis revealed 1842 consistently differentially expressed genes (DEGs) across both time points, including 660 persistently upregulated and 1182 persistently downregulated genes (Table S3). These DEGs were likely associated with the peach defense response to BS disease. Within the list of up-regulated genes, we identified multiple pathogenesis-related (PR) genes that exhibited significant induction at 2 dpi, including glycosyl hydrolases (PR2), thaumatin (PR5), and peroxidase (PR9) family genes (Fig. S1A). Concurrently, phytohormone profiling revealed a gradual increase in the level of SA, a key defense-related hormone, following pathogen inoculation (Fig. S1B). These observations collectively suggest the activation of a robust defense response at the site of pathogen inoculation.

Given that phenylpropanoid-derived secondary metabolites are well known for being vital in plant disease resistance, the expression of phenylpropanoid pathway genes was analyzed. Transcriptome data revealed strong upregulation of key genes in the PA biosynthetic pathway at 2 dpi (Fig. 1B), suggesting PA accumulation in response to pathogenic inoculation. To test this, pathogen inoculation was conducted in leaves and fruits of the peach cultivar ‘XHJ’, which has been widely documented in the literature as a highly suitable system for transient genetic transformation studies [39–41]. Pathogen-inoculated tissues showed significantly greater lesion formation than mock-treated controls (Fig. 1C and G). DMACA staining and spectrophotometric analysis confirmed substantial PA accumulation in both leaves and fruits (Fig. 1D, E, H, I). Additionally, qRT-PCR analysis verified the expression levels of *PpANR* and *PpLAR*, which has been reported playing critical roles in PA biosynthesis [42, 43], showing coordinated upregulation in pathogen-inoculated tissues (Fig. 1F and J). Together, these findings provided strong evidence that PA specifically accumulated in peach under pathogenic bacterial inoculation.

### PA mitigates the severity of disease symptoms induced by pathogenic bacteria

Plants subjected to abiotic stress or pathogen inoculation accumulated excessive ROS, known to trigger cell death [2, 6]. To investigate this, we performed the DAB staining to detect H₂O₂, a well-characterized ROS, in peach leaves and fruits inoculated with the pathogen. Compared to the H_2_O-treated controls, pathogen inoculation significantly induced H₂O₂ accumulation (Fig. S2A and B). We subsequently treated peach leaves and fruits with 10 mM H_2_O_2_ and found H_2_O_2_-treated tissues exhibited phenotypes resembling those of pathogen-inoculated tissues, including pronounced cell death (Fig. S2C and D). Fruits developed more extensive necrosis than leaves at the same H_2_O_2_ concentration, indicating that ROS overaccumulation induced cell death.

**Figure 2:**
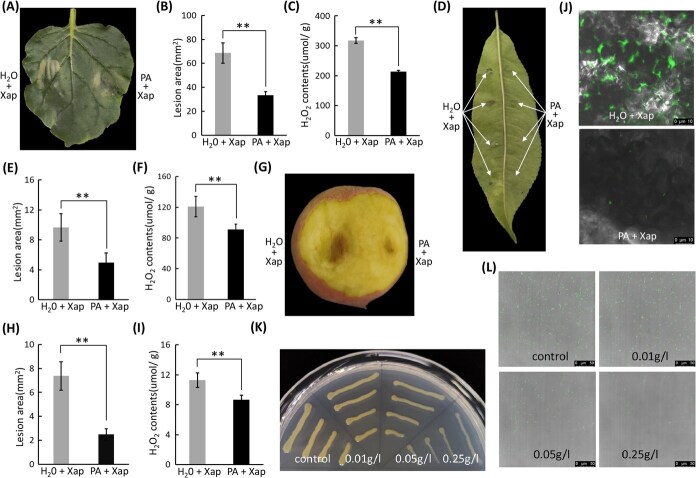
PA mitigates disease severity induced by pathogenic bacterial infection. (A) Effect of exogenous PA treatment on pathogen-inoculated tobacco leaves. (B) Impact of exogenous PA treatment on lesion area in pathogen-inoculated tobacco leaves. (C) Exogenous PA treatment modulates H_2_O_2_ levels in pathogen-inoculated tobacco leaves. (D) Effect of exogenous PA treatment on pathogen-inoculated peach leaves. (E) Impact of exogenous PA treatment on lesion area in pathogen-inoculated peach leaves. (F) Exogenous PA treatment modulates H_2_O_2_ levels in pathogen-inoculated peach leaves. (G) Effect of exogenous PA treatment on pathogen-inoculated peach fruits. (H) Impact of exogenous PA treatment on lesion area in pathogen-inoculated peach fruits. (I) Exogenous PA treatment modulates H_2_O_2_ levels in pathogen-inoculated peach fruits. (J) Exogenous PA application modulates fluorescence intensity of GFP-labeled pathogens in inoculated tissues. (K) Exogenous PA application affects pathogen growth under *in vitro* culture conditions. (L) Exogenous PA application reduces the number of GFP-labeled pathogens under *in vitro* conditions. Error bars in (B), (C), (E), (F), (H), and (I) represent the SE of three biological replicates.

As PA exhibit strong antioxidant activity and neutralize ROS, such as superoxide anions and H_2_O_2_, it was hypothesized that PA accumulation contributed to disease resistance via ROS scavenging. To test this, co-infiltration experiments with exogenous PA and pathogen were performed in tobacco leaves. PA treatment significantly reduced both the severity (Fig. 2A) and area (Fig. 2B) of necrotic lesions, along with reduced H_2_O_2_ levels in the treated leaves (Fig. 2C). Similar effects were observed in peach leaves, with reduced symptoms (Fig. 2D), necrotic area (Fig. 2E), and H_2_O_2_ content (Fig. 2F). Consistent results were obtained in peach fruits (Fig. 2G–I). To assess whether PA directly affected pathogen growth, GFP-labeled pathogens were monitored, and PA application significantly reduced fluorescence intensity in inoculated tissues (Fig. 2J). *In vitro* assays further demonstrated a dose-dependent inhibition of pathogenic bacterial growth (Fig. 2K and L). These findings indicated that PA mitigated disease development by scavenging ROS and suppressing pathogen proliferation.

### *PpMYB123* plays a crucial role for PA accumulation

**Figure 3:**
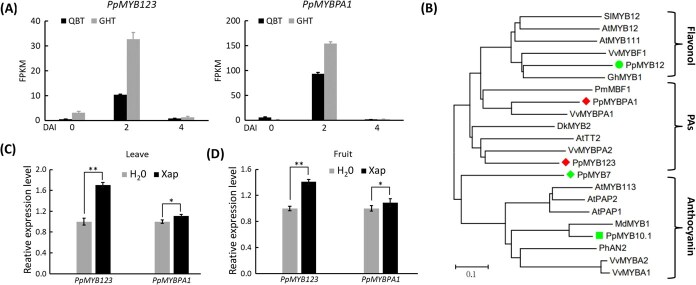
Expression analysis and identification of the *PpMYB123* and *PpMYBPA1* genes. (A) Transcriptional profiles of *PpMYB123* and *PpMYBPA1* in peach leaves following pathogen inoculation. (B) Phylogenetic analysis of *PpMYB123* and *PpMYBPA1* in peach and other plant species. Peach genes are highlighted with colored blocks. Sequences were retrieved from the NCBI database with the following accessions: *Prunus persica* PpMYB7 (XP_007210006.2), PpMYB12 (ONH94094.1), PpMYB123 (XP_007224456.1), and PpMYBPA1 (XP_007218760.1); *Arabidopsis thaliana* AtMYB12 (NP182268), AtMYB111 (NP182268), AtTT2 (NP_198405.1), AtMYB113 (NP_176811.1), AtPAP1 (ABB03879.1), and AtPAP2 (NP_176813.1); *Vitis vinifera* VvMYBF1 (NP_176813.1), VvMYBPA1 (CAJ90831.1), VvMYBPA2 (ACK56131.1), VvMYBA1 (BAD18977.1), and VvMYBA2 (BAD18978.1); *Diospyros kaki* DkMYB2 (BAI49719.1); *Picea mariana* PmMBF1 (AAA82943); *Malus domestica* MdMYB1 (ABK58136.1); *Petunia × hybrida* PhAN2 (AAF66727.1); *Solanum lycopersicum* SIMYB12 (NP001234401.1); and *Gerbera hybrid* GhMYB1 (CAD87007). (C) Expression patterns of *PpMYB123* and *PpMYBPA1* in peach leaves under pathogen inoculation. (D) Expression patterns of *PpMYB123* and *PpMYBPA1* in peach fruits under pathogen inoculation. Error bars in (A), (C), and (D) indicate the standard error (SE) of three biological replicates.

In the list of MYB transcription factors upregulated at 2 dpi (Table S4), two MYB genes, *PpMYB123* (Prupe.1G405400) and *PpMYBPA1* (Prupe.2G192100), were identified, both exhibiting expression patterns similar to *PpANR* and *PpLAR* (Fig. 3A). Correlation analysis revealed a significant association between their expression profiles and those of structural genes involved in PA biosynthesis regulation (Fig. S3). Phylogenetic analysis placed these two genes in a distinct clade, separate from MYB regulators of flavonol and anthocyanin biosynthesis, and confirmed their orthology with *AtTT2*, the master regulator of PA biosynthesis in Arabidopsis (Fig. 3B). Expression analysis in peach leaves (Fig. 3C) and fruits (Fig. 3D) inoculated with pathogenic bacteria showed that both genes were upregulated, with *PpMYB123* displaying more pronounced variation, suggesting a key role in PA accumulation under pathogen infection.

Functional validation in tobacco leaves (Fig. S4A) showed that overexpression of *PpMYB123* significantly increased PA accumulation (Fig. S4B and C) and upregulated *NtANR* and *NtLAR* (Fig. S4D). Transient overexpression in peach leaves also promoted PA accumulation (Fig. S4E–G) and significantly upregulated *PpANR* and *PpLAR* (Fig. S4H). Similar results were observed in peach fruits (Fig. S4I–L). These findings indicated that *PpMYB123* played a central role in PA accumulation.

### PpMYB123-induced PA accumulation alleviates the disease symptoms under pathogenic bacteria inoculation

**Figure 4:**
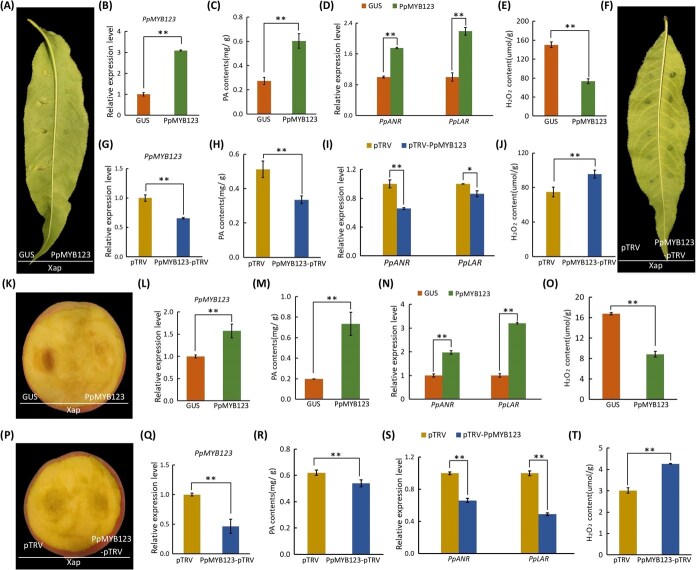
*PpMYB123*-induced PA accumulation alleviates disease symptoms under pathogen inoculation. (A) Phenotypes of *PpMYB123*-overexpressing and control peach leaves following pathogen inoculation; *GUS* overexpression was used as the control. (B) *PpMYB123* expression levels in overexpressing and control peach leaves. (C) PA contents in *PpMYB123*-overexpressing and control peach leaves. (D) Transcriptional levels of *PpANR* and *PpLAR* in *PpMYB123*-overexpressing and control peach leaves. (E) H_2_O_2_ contents in *PpMYB123*-overexpressing and control peach leaves. (F) Phenotypes of *PpMYB123*-silenced and control peach leaves under pathogen inoculation; the empty pTRV2 vector was used as the control. (G) *PpMYB123* expression levels in silenced and control peach leaves. (H) PA contents in *PpMYB123*-silenced and control peach leaves. (I) Transcriptional levels of *PpANR* and *PpLAR* in *PpMYB123*-silenced and control peach leaves. (J) H_2_O_2_ contents in *PpMYB123*-silenced and control peach leaves. (K) Phenotypes of *PpMYB123*-overexpressing and control peach fruits following pathogen inoculation; *GUS* overexpression was used as the control. (L) *PpMYB123* expression levels in overexpressing and control peach fruits. (M) PA contents in *PpMYB123*-overexpressing and control peach fruits. (N) Transcriptional levels of *PpANR* and *PpLAR* in *PpMYB123*-overexpressing and control peach fruits. (O) H_2_O_2_ contents in *PpMYB123*-overexpressing and control peach fruits. (P) Phenotypes of *PpMYB123*-silenced and control peach fruits under pathogen inoculation; the empty pTRV2 vector was used as the control. (Q) *PpMYB123* expression levels in silenced and control peach fruits. (R) PA contents in *PpMYB123*-silenced and control peach fruits. (S) Transcriptional levels of *PpANR* and *PpLAR* in *PpMYB123*-silenced and control peach fruits. (T) H_2_O_2_ contents in *PpMYB123*-silenced and control peach fruits. Error bars represent the standard error (SE) of three biological replicates.

To further elucidate the pivotal role of *PpMYB123* in mitigating pathogen-induced damage, the functional validation approach was employed. In peach leaves, transient overexpression of *PpMYB123* led to a significant reduction in lesion size (Fig. 4A and B), accompanied by a marked increase in PA content (Fig. 4C), significant upregulation of *PpANR* and *PpLAR* expression (Fig. 4D), and a notable reduction in H_2_O_2_ content (Fig. 4E). Conversely, silencing of *PpMYB123* exacerbated cell death, increased lesion size (Fig. 4F and G), reduced PA content by ~30% (Fig. 4H), significantly downregulated PA biosynthetic genes (Fig. 4I), and elevated H_2_O_2_ levels (Fig. 4J). Further functional studies of *PpMYB123* in peach fruits yielded results consistent with those observed in peach leaves. Overexpression of *PpMYB123* mitigated pathogen-induced damage (Fig. 4K and L), resulted in a threefold increase in PA content (Fig. 4M), upregulated *PpANR* and *PpLAR* expression by at least twofold (Fig. 4N), and reduced H_2_O_2_ content by approximately 50% (Fig. 4O). Conversely, *PpMYB123* silencing produced an opposite phenotype, significantly exacerbating disease progression (Fig. 4P–T). Therefore, these findings collectively indicated that PpMYB123-induced PA accumulation alleviated pathogen-induced damage in both peach leaves and fruits.

### PpMYB123 directly promotes the expression of *PpANR* and *PpLAR*

**Figure 5:**
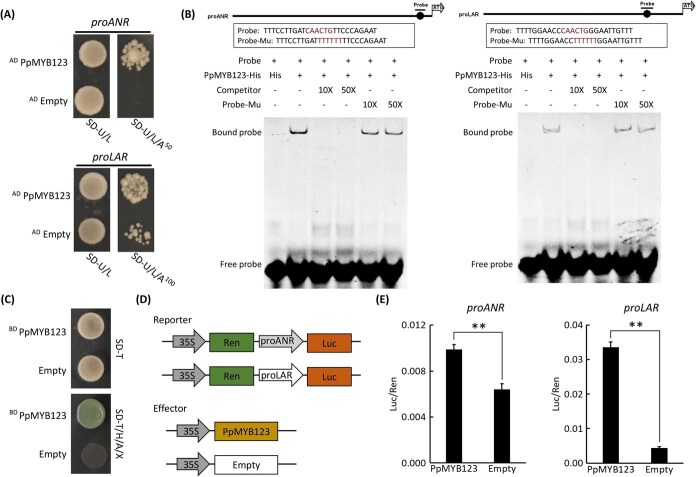
PpMYB123 directly promotes the expression of *PpANR* and *PpLAR*. (A) Assessment of the binding ability of PpMYB123 to the *PpANR* and *PpLAR* promoters using Y1H. (B) Assessment of the binding ability of PpMYB123 to the MBS in the promoters of *PpANR* and *PpLAR* using EMSA. (C) Transcriptional activity analysis of the *PpMYB123* gene in yeast. (D) Schematic representation of reporter and effector constructs used in the dual-luciferase assay. (E) Evaluation of PpMYB123-mediated transcriptional activation of *PpANR* and *PpLAR* promoters using the dual-luciferase assay. Error bars represent the SE of three biological replicates.

Previous studies had established that MYB transcription factors directly regulated PA biosynthesis by activating *AN*R and *LAR* gene expression [33, 34, 37]. Based on this, it was hypothesized that PpMYB123 might employ a similar mechanism. Yeast one-hybrid (Y1H) assays showed that PpMYB123 physically interacted with the promoter regions of *PpANR* and *PpLAR* (Fig. 5A). Cis-element analysis identified conserved MYB-binding sites (MBS, ‘CAACA/TG’) and MYB recognition sequences (MRS, ‘CCGTTG’) within their promoters (Fig. S5A). Electrophoretic mobility shift assay (EMSA) confirmed specific binding of PpMYB123 to an MBS motif (‘CAACTG’) near the start codon (Fig. S5B). Site-directed mutagenesis showed that substituting the core MBS sequence with ‘TTTTTT’ abolished the binding ability of PpMYB123, confirming the critical role of these nucleotides in mediating protein–DNA interaction (Fig. 5B). Functional assays in yeast demonstrated strong transactivation activity of PpMYB123, with its activation domains driving reporter gene expression (Fig. 5C). Dual-luciferase assays further confirmed that PpMYB123 significantly enhanced transcriptional activation of both *PpANR* and *PpLAR* promoters (Fig. 5D). These results collectively demonstrated that PpMYB123 directly bound to the promoters of *PpANR* and *PpLAR*, promoting their transcription.

### PpPUB23 negatively regulates the function of PpMYB123

**Figure 6:**
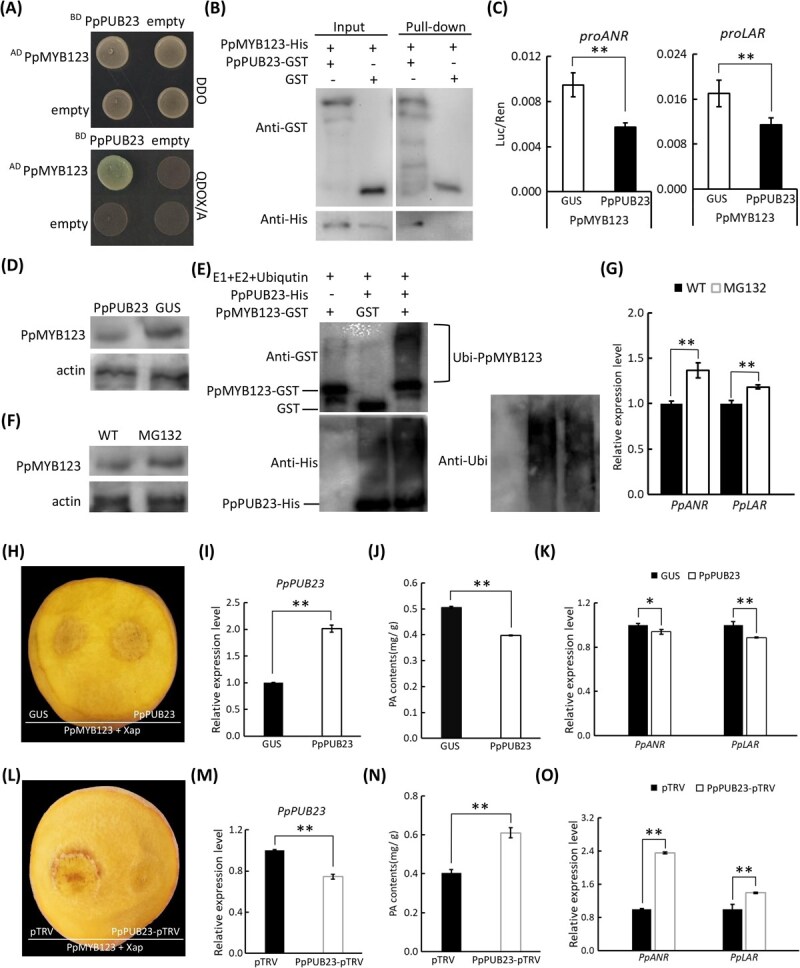
PpPUB23 negatively regulates the function of PpMYB123. (A) Validation of the interaction between PpMYB123 and PpPPUB23 using Y2H. (B) Validation of the interaction between PpMYB123 and PpPUB23 using GST pull-down. (C) Assessment of the effect of PpPUB23 on PpMYB123-mediated transcriptional activation of *PpANR* and *PpLAR* promoters using the dual-luciferase assay. (D) Analysis of PpMYB123 protein levels in PpPUB23-overexpressing and control peach fruit. (E) An *in vitro* ubiquitination assay was employed to demonstrate that PpPUB23 mediates the ubiquitination of PpMYB123. The reaction products were detected using anti-GST, anti-ubiquitin, and anti-His antibodies. (F) Impact of proteasome inhibitor MG-132 on the abundance of PpMYB123 protein. (G) Effect of proteasome inhibitor MG-132 on the expression of *PpANR and PpLAR*. (H) Phenotypes of *PpPUB23*-overexpressing and control peach fruits following pathogen inoculation; *GUS* overexpression served as the control. (I) Expression levels of *PpPUB23* in *PpPUB23*-overexpressing and control peach fruits. (J) PA content in *PpPUB23*-overexpressing and control peach fruits. (K) Transcriptional levels of *PpANR* and *PpLAR* in *PpPUB23*-overexpressing and control peach fruits. (L) Phenotypes of *PpPUB23*-silenced and control peach fruits under pathogen inoculation; empty pTRV2 vector was used as the control. (M) Expression levels of *PpPUB23* in *PpPUB23*-silenced and control peach fruits. (N) PA content in *PpPUB23*-silenced and control peach fruits. (O) Transcriptional levels of *PpANR* and *PpLAR* in *PpPUB23*-silenced and control peach fruits. Error bars in (C), (G), (I), (J), (K), (M), (N), and (O) represent the SE of three biological replicates.

To investigate potential post-translational modifications (PTMs) of PpMYB123, we performed a yeast two-hybrid (Y2H) screening and identified *PpPUB23*, a U-box-type E3 ubiquitin ligase. Y2H and GST pull-down assays confirmed a direct interaction between PpPUB23 and PpMYB123 (Fig. 6A and B). Phylogenetic analysis identified *PpPUB23* as an ortholog of Arabidopsis *AtPUB23*, clustering with homologs from sweet cherry, apple, and pear (Fig. S6A). Domain analysis confirmed a canonical U-box domain at the N-terminus of PpPUB23 (Fig. S6B). Surprisingly, transcriptome profiling revealed significant downregulation of *PpPUB23* upon pathogenic bacterial inoculation (Fig. S6C). Dual-luciferase assays showed that PpPUB23 substantially reduced the transcriptional activation of *PpANR* and *PpLAR* by PpMYB123 (Fig. 6C), suggesting its role in PpMYB123 degradation via the ubiquitination pathway. To investigate whether PpPUB23 influences the stability of the PpMYB123 protein, we examined the abundance of PpMYB123 protein in *PpPUB23*-overexpressing and control peach fruits. The results showed that overexpression of *PpPUB23* led to a significant reduction in PpMYB123 protein levels compared to the *GUS* control group (Fig. 6D). *In vitro* ubiquitination assays further demonstrated that the PpMYB123-GST fusion protein, but not the GST-tagged control, underwent PpPUB23-mediated ubiquitination and subsequent degradation (Fig. 6E). Additionally, treatment with the proteasome inhibitor MG-132 resulted in an accumulation of PpMYB123 protein in peach fruits (Fig. 6F), which was accompanied by a significant up-regulation of PA biosynthetic genes, *PpANR* and *PpLAR* (Fig. 6G). Collectively, these results indicate that PpPUB23 modulates PA biosynthesis by facilitating the ubiquitin-dependent degradation of PpMYB123.

Functional validation in peach fruits was performed, showing that overexpression of *PpPUB23* led to increased cell death at inoculation sites (Fig. 6H and I), reduced PA accumulation (Fig. 6J), and suppressed expression of *PpANR* and *PpLAR* (Fig. 6K). Conversely, silencing of *PpPUB23* reduced lesion size (Fig. 6L and M), enhanced PA accumulation (Fig. 6N), and upregulated *PpANR* and *PpLAR* expression (Fig. 6O). These complementary results established that PpPUB23 negatively regulated PpMYB123, likely via ubiquitin–proteasome-mediated post-translational regulation, thereby fine-tuning PA-mediated defense responses in peach.

## Discussion

### PA mitigates pathogen-induced damage through removing excessive ROS and suppressing pathogen growth in peach

Secondary metabolites, such as lignin, flavonol, anthocyanin, and PA, produced via the phenylpropanoid pathway, were reported to contribute to plant disease resistance [14–21]. In this study, the expression of PA biosynthetic genes *PpANR* and *PpLAR* was specifically upregulated in peach leaves following inoculation by the peach BS pathogen (Fig. 1A and B), leading to PA accumulation (Fig. 1C–E), which was also observed in peach fruits (Fig. 1G–J). Exogenous PA application significantly reduced pathogen-induced cell death in tobacco leaves, peach leaves, and fruits (Fig. 2A, D, G), establishing PA as a defense compound. These findings aligned with previous reports on PA-mediated resistance to powdery mildew in grapevine and fungal disease in poplar [20, 22–25], suggesting a conserved PA-based defense mechanism across plant species.

ROS were identified as key signaling molecules in plant stress responses, contributing to resistance against biotic and abiotic stresses and supporting essential physiological functions [5, 44, 45]. However, excessive ROS during the hypersensitive response (HR) caused oxidative damage and cell death [2, 6]. In this study, pathogen inoculation and high-concentration H_2_O_2_ treatment induced pronounced necrosis in peach leaves and fruits (Fig. 1C and G; Fig. S2), confirming ROS overaccumulation as a key driver of cellular damage, consistent with findings in rice and cucumber [46, 47].

Given PA's exceptional antioxidant capacity, it was hypothesized that PA could mitigate pathogen-induced damage by removing excess ROS. Experimental validation in tobacco leaves, peach leaves, and peach fruits showed that PA treatment significantly reduced H_2_O_2_ levels at inoculated sites (Fig. 2C, F, I). This ROS-scavenging activity was attributed to PA's unique molecular structure [17–21]. Additionally, both *in planta* and *in vitro* assays revealed that PA directly inhibited pathogenic bacterial growth (Fig. 2J and K), possibly due to the combined effect of PA and lignin in strengthening cell walls [22, 23]. In conclusion, it was demonstrated for the first time that PA mitigated pathogen-induced damage in peach by scavenging ROS and directly inhibiting pathogen growth.

### Pathogen-induced PpMYB123 activates PA biosynthesis via transcriptional activation of *PpANR* and *PpLAR*

The expression of PA biosynthetic genes was primarily regulated by upstream transcription factors, with MYB family members playing a key role. In Arabidopsis, the MYB transcription factor encoded by *AtTT2* directly activated *AtANR*/*BAN* transcription to promote PA accumulation in seed coats [33]. In fruit crops, MYB regulators identified included *VvMYBPA1*, *VvMYB5b*, and *VvMYBPA2* in grape [34–36], and *FaMYB9*/*FaMYB11* and *FaMYB5* in strawberry [37, 38]. In peach, MYB genes such as *MYBPA1*, *MYB7*, and *Peace*/*PpMYB6* were reported to regulate PA accumulation [43, 48, 49]. However, the specific MYBs involved in pathogen-induced defense responses remained unclear.

In this study, we identified two MYB transcription factors, *PpMYB123* and *PpMYBPA1*, from the set of MYBs upregulated at 2 dpi. In contrast to the downregulation of *PpMYB7* and the near absence of *Peace*/*PpMYB6* expression, the expression patterns of *PpMYB123* and *PpMYBPA1* were strongly correlated with those of the PA biosynthetic genes *PpANR* and *PpLAR* (Fig. 3A). Phylogenetic analysis revealed that both genes are homologs of Arabidopsis *AtTT2* (Fig. 3B). Notably, compared to *PpMYBPA1*, *PpMYB123* showed more pronounced upregulation in both peach leaves and fruits following pathogen inoculation (Fig. 3C). Overexpression of *PpMYB123* significantly enhanced PA accumulation in tobacco, as well as in peach leaves and fruits (Fig. S4). In peach leaves and fruits, *PpMYB123* overexpression enhanced disease resistance, accompanied by increased PA accumulation and significantly reduced H_2_O_2_ levels. Conversely, silencing *PpMYB123* aggravated cell death symptoms, reduced PA biosynthesis, and elevated H_2_O_2_ accumulation (Fig. 4). These findings demonstrate that PpMYB123 confers resistance to BS disease by promoting PA accumulation in peach.

The Y1H, EMSA, and Luc/Ren assays collectively demonstrated that PpMYB123 directly binds to the *PpANR* and *PpLAR* promoters (Fig. 5). Further investigation revealed that PpMYB123 specifically recognizes and binds to a DNA fragment containing the MBS motif ‘CAACTG’, located proximal to the ATG start codon (Fig. S5), suggesting evolutionary conservation in its DNA-binding site. Transcriptomic analysis showed coordinated upregulation of early biosynthetic genes (EBGs) shared by the anthocyanin, flavonol, and PA pathways, indicating that PpMYB123 may also regulate these common EBGs. Notably, unlike the expression pattern of *PpANR* and *PpLAR*, the genes controlling anthocyanin and flavonol biosynthesis were significantly downregulated following pathogen inoculation (Fig. 1B). This distinct expression pattern strongly suggests that metabolic flux is preferentially directed toward PA biosynthesis at the expense of anthocyanin and flavonol production.

### PpPUB23–PpMYB123 module-mediated PA accumulation as an immune response to BS disease in peach

PTMs, including phosphorylation and ubiquitination, are crucial for regulating protein stability and function [50–53]. Plant U-box type E3 ubiquitin ligases (PUBs), characterized by their conserved U-box domain and E3 ubiquitin ligase activity, regulate various biological processes, including development and stress responses [54–57]. Recent studies have shown that PUB proteins play a role in plant disease resistance [25, 58–60], but their functions in peach remain unexplored. In this study, we identified PpPUB23, a PUB family protein that interacts with PpMYB123, through Y2H screening. Biochemical assays confirmed their direct physical interaction *in vitro* (Fig. 6B). Functional characterization revealed that PpPUB23 attenuates the transcriptional activation of *PpANR* and *PpLAR* by PpMYB123 (Fig. 6C). Results from the assays *in vivo*, *in vitro*, and MG-132 treatment in peach fruits further demonstrate that PpPUB23 modulates the transcriptional activation of PA biosynthetic genes by PpMYB123 through ubiquitin-mediated regulation of its protein stability (Fig. 6D–G). *PpPUB23* overexpression reduced PA accumulation in peach fruits, whereas its silencing enhanced PA content (Fig. 6H–O), demonstrating its negative regulatory role in PpMYB123-mediated PA biosynthesis. Notably, pathogen inoculation significantly downregulated *PpPUB23* expression (Fig. S6B), presumably relieving its suppression of PpMYB123 and facilitating PA accumulation during defense responses. Pathogen inoculation induced localized cell necrosis at inoculation sites (Fig. 1A), accompanied by up-regulation of PR genes, a marked increase in the level of the defense hormone SA (Fig. S1), and substantial accumulation of ROS (Fig. S2), which represent hallmark manifestations of immune response in plants [61–64]. This study demonstrates that PA accumulation after pathogen inoculation alleviates disease severity, indicating that PA biosynthesis constitutes a defense response. These findings imply that the PpPUB23–PpMYB123 regulatory module-mediated PA accumulation represents a functional component of immune response against BS disease in peach.

**Figure 7:**
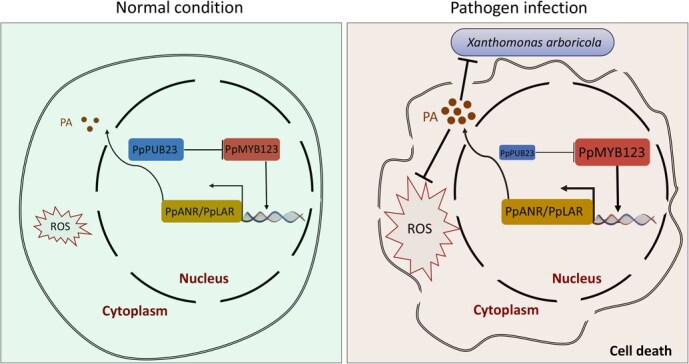
A proposed model of *PpMYB123-*mediated PA accumulation alleviating BS disease in peach. Under normal conditions (left), basal ROS levels prevent oxidative cytotoxicity, while the expression of *PpMYB123* and its inhibitory E3 ligase *PpPUB23* remains at constitutive levels, resulting in steady-state PA accumulation. In contrast, pathogen inoculation (Right) induces excessive ROS production in peach leaves and fruits, leading to oxidative damage and cell death. The pathogen inoculation upregulates *PpMYB123* expression, which consequently activates transcription of PA biosynthetic genes *PpANR* and *PpLAR*, thereby enhancing PA accumulation at inoculated tissues. Concurrently, the expression of *PpPUB23*, a negative regulator of PpMYB123, is suppressed, further amplifying PpMYB123-mediated PA biosynthesis. PA contributes to disease resistance by both scavenging excess ROS to reduce cell death and directly inhibiting pathogen growth, ultimately mitigating the BS disease incidence in peach.

Based on these findings, we propose a molecular mechanism by which PpMYB123-mediated PA accumulation alleviates BS disease in peach (Fig. 7). Under normal conditions (left), basal ROS levels prevent oxidative cytotoxicity, with the expression of *PpMYB123* and its inhibitory E3 ligase *PpPUB23* maintaining steady-state PA accumulation. Upon pathogen inoculation, excessive ROS production in peach leaves and fruits tissues leads to oxidative damage and cell death (right). Pathogen inoculation upregulates *PpMYB123* expression PA biosynthetic genes *PpANR* and *PpLAR* and promoting PA accumulation. Concurrently, pathogen inoculation suppresses the expression of *PpPUB23* (the negative regulator of PpMYB123), thereby amplifying PpMYB123’s regulatory effect on PA accumulation. PA reduces cell death by scavenging excessive ROS and directly inhibits pathogen growth, ultimately alleviating BS disease in peach.

## Materials and methods

### Plant materials and growth conditions

Leaves of the wild peach variety ‘Guanghetao’ (GHT) (*Prunus mira*) and the cultivated varieties ‘Qiubaitao’ (QBT) and ‘Xiahuangjin’ (XHJ) (*Prunus persica*) were collected from the fourth to seventh newly formed leaves at the tops of branches. Fruits of XHJ were harvested at the early ripening stage. All cultivars were maintained at the Wuhan Botanical Garden, Chinese Academy of Sciences, Wuhan, China. The collected leaves and fruits were rinsed with water, disinfected with 75% alcohol, and inoculated with *Xanthomonas arboricola* pv. *pruni* (Xap), isolated from peach leaves showing BS disease, using the sterile syringe without the needle and the sterile syringe with the needle respectively according to previous reports [65, 66]. Inoculated samples were incubated at 25°C with 60% humidity under a 16-h/8-h day/night cycle. After collection, samples were immediately frozen in liquid nitrogen and stored at −80°C. Additionally, *Nicotiana benthamiana* seedlings were cultivated in a controlled chamber as previously described [40].

### Quantifying of PA and H_2_O_2_

The PA content was determined following Zhao *et al*. [25]. Approximately 1 g of ground sample was placed in a 1.5-ml tube, mixed with 1 ml of 60% methanol, and incubated at 4°C in the dark for 30 min. After centrifugation, 600 μl of supernatant was transferred to a new tube and mixed with 300 μl of DMACA solution (0.1 g DMACA in 100 ml of 1 M HCl in methanol). After 30 min at room temperature, absorbance was measured at 640 nm using an enzyme-linked immunosorbent assay (ELISA) reader (TECAN Infinite M200, Austria). DMACA staining followed a lab-established method [67], and images were captured with a digital camera. Hydrogen peroxide (H_2_O_2_) was extracted and quantified using an H_2_O_2_ detection kit (Solarbio Science & Technology, Beijing, China). 3,3′-diaminobenzidine (DAB) staining was performed according to previous study [25]. All experiments included three biological replicates per sample.

### Extraction of total RNA and quantitative RT-PCR (qRT-qPCR) analysis

RNA extraction and quantitative real-time PCR (qRT-PCR) experiments were conducted following protocols from a previous study [41]. Approximately 100 mg of samples, ground in liquid nitrogen, were used for total RNA extraction with the HiPure HP Plant RNA Mini Kit (Magen, Guangzhou, China). The extracted RNA was reverse-transcribed into cDNA using the PrimerScript™ RT Reagent Kit with gDNA Eraser (Yeasen, Shanghai, China). Gene expression levels were quantified using the Applied Biosystems™ 7300 Real-Time PCR System (USA), and relative expression was calculated using the 2^−ΔΔCT^ method, with *PpGADPH* as the internal reference [39]. Three biological replicates were included per sample. Primer sequences are listed in Table S1.

### Library construction and transcriptome analysis

RNA concentration and integrity were assessed using the NanoDrop 2000 spectrophotometer (Thermo Fisher Scientific, USA) and Agilent 2100 Bioanalyzer (Agilent Technologies, USA). cDNA libraries were constructed with the CloneMiner™ cDNA Library Construction Kit (Thermo Fisher Scientific, Waltham, USA), and paired-end sequencing was performed by Beijing Biomarker Technologies Co., LTD. on the Illumina HiSeq Xten high-throughput sequencing platform. Raw sequencing data were quality-checked, including removal of low-quality reads and adapter sequences, and aligned to the peach reference genome [68] using HISAT2 and Bowtie. Gene expression levels were normalized and quantified using FPKM (Fragments Per Kilobase of transcript per Million mapped reads).

### Phylogenetic analysis and protein sequence alignment

Based on the *AtMYB111*, *AtMYB12*, *AtTT2*, *AtPAP1*, and *AtPAP2* protein sequences from *Arabidopsis thaliana*, homologous genes in phylogenetically diverse species, including *Vitis vinifera*, *P. persica*, and *Malus domestica*, were identified using the National Center for Biotechnology Information (NCBI) database. Retrieved sequences were aligned using CLUSTALX to assess conservation and variability. A phylogenetic tree was reconstructed in MEGA6 using maximum likelihood or neighbor-joining methods to infer evolutionary relationships among these genes across the examined species. Bootstrap analysis with 1000 replicates was conducted to evaluate the robustness of the tree topology. The same approach was applied for *PUB* genes.

### Yeast one-hybrid and electrophoretic mobility shift assay

The Y1H assay was performed as described previously [40]. Briefly, the *PpMYB123* coding sequence (CDS) was amplified from ‘GHT’ cDNA and ligated into the pGADT7 vector. Promoter regions of *PpANR* and *PpLAR* were amplified from ‘GHT’ genomic DNA and cloned into the pAbAi vector. The recombinant plasmids were co-transformed into the Y1H yeast strain. Protein–DNA interactions between PpMYB123 and the *PpANR* and *PpLAR* promoters were validated by selective screening on nutrient-deficient media containing specific Aureobasidin A (AbA) concentrations.

The EMSA experiment was conducted according to the established protocol [69]. Specifically, the *PpMYB123* CDS was amplified and cloned into the pET32a expression vector, which was subsequently transformed into *Escherichia coli* BL21(DE3) competent cells. The His-tagged recombinant protein was expressed under isopropyl β-d-1-thiogalactopyranoside (IPTG) induction and purified using nickel-affinity chromatography. The target DNA probes were labeled with 5-carboxyfluorescein (5-FAM). Experimental conditions were optimized following the manufacturer's instructions for the Chemiluminescent EMSA Kit (Beyotime, Shanghai, China). The purified PpMYB123 protein was incubated with the labeled probes in binding buffer for 30 min at room temperature. The reaction mixtures were resolved on a 6% non-denaturing polyacrylamide gel. Following electrophoresis, the gel was visualized using a multifunctional laser imaging system (Leica, Wetzlar, Germany) to detect protein–DNA complexes based on fluorescence signals. The primers used for vector construction are listed in Table S2.

### Y2H and GST pull-down assay

The Y2H screening assay was conducted in accordance with established protocols described in a previous study [39], using a yeast cDNA library derived from peach fruit tissues. Candidate genes identified through primary screening were amplified by PCR using the ‘GHT’ cDNA as the template and directionally cloned into the pGBKT7 bait vector. The resulting recombinant bait vectors were co-transformed with the AD-PpMYB123 prey plasmid into the Y2HGold yeast strain. To validate protein–protein interactions, the transformed yeast cells were plated onto selective media, including double dropout (DDO; SD/–Trp/–Leu) and quadruple dropout (QDO/X/A; SD/–Trp/–Leu/–His/–Ade supplemented with X-α-gal and Aureobasidin A) agar plates, and incubated at 30°C for 3 days to assess colony growth and color development.

The GST pull-down assay was performed according to the methods described in a previous study [40]. The CDS of *PpPUB23* was amplified from the cDNA of ‘GHT’ and subsequently cloned into the pGEX-4T expression vector. The recombinant plasmid was then transformed into *E. coli* BL21 competent cells, and the PpPUB23-GST fusion protein expression was induced by IPTG. The fusion protein was purified using glutathione-sepharose bead affinity chromatography and subsequently co-incubated with the PpMYB123-His fusion protein for *in vitro* interaction assays. Protein complexes were isolated via GST affinity purification and separated by SDS-PAGE. Immunoblotting was conducted using monoclonal antibodies specific to GST and His epitopes to confirm the physical interaction between the two recombinant proteins. The primers used for vector construction are listed in Table S2.

### Dual luciferase expression assay

The *PpANR* and *PpLAR* promoter regions were amplified from the ‘GHT’ DNA and cloned into the pGreenII 0800-LUC binary vector. The recombinant constructs were electroporated into *Agrobacterium tumefaciens* strain GV3101 harboring the pSoup-p19 helper plasmid. Simultaneously, the CDSs of *PpMYB123* and *PpPUB23* were amplified from the ‘GHT’ cDNA and directionally cloned into the pSAK277 expression vector using restriction enzymes. The resulting constructs were introduced into *A. tumefaciens* GV3101 competent cells via electroporation. For transient expression assays, Agrobacterium cultures carrying the different constructs were co-infiltrated into the abaxial surface of fully expanded leaves of 3-week-old tobacco plants, following the optimized protocol described in a previous study [39]. At 48 h post-infiltration (hpi), leaf discs were harvested and homogenized in Passive Lysis Buffer. The luminescent signals from firefly luciferase (Luc) and Renilla luciferase (Ren) were quantified using the Dual-Luciferase Reporter Gene Assay Kit (Yeasen, Shanghai, China), following the manufacturer’s instructions. To normalize for transformation efficiency, relative luciferase activity was calculated as the ratio of Luc to Ren. The primers used for vector construction are listed in Table S2.

### *In vivo* protein degradation assay

The *in vivo* protein degradation assay was performed according to previously established methods [70]. Total proteins were extracted from peach fruits subjected to transient gene overexpression and treatment with the proteasome inhibitor MG132 (50 μM) using a commercial protein extraction kit (Solarbio, Beijing, China). The extracted proteins were separated by SDS-PAGE and subsequently transferred onto PVDF membranes. The membranes were incubated with the PpMYB123-specific primary antibody (Mabstar, Wuhan, China; dilution 1:2000) or the anti-actin antibody (Mabstar, Wuhan, China; dilution 1:2000), followed by detection with the horseradish peroxidase (HRP)-conjugated secondary antibody. Chemiluminescent imaging was carried out using a multifunctional imaging system (FluorChem R, ProteinSimple, USA).

### *In vitro* ubiquitination assay

The *in vitro* ubiquitination assay was conducted based on an established methodology [70]. The constructed fusion expression vectors PpPUB23-His and PpMYB123-GST were transformed into BL21 strain, and protein expression was induced with IPTG. A 100-μl reaction mixture was prepared containing 2.5 μl of 20× Ubiquitin Activating Enzyme (E1) Solution, 5 μl of 10× Ubiquitin Conjugating Enzyme (E2, human UbcH5b) Solution, 2.5 μl of 20× Mg-ATP Solution, 5 μl of 10× Ubiquitinylation Buffer, along with purified PpPUB23-His and PpMYB123-GST proteins. The reaction was incubated at 37°C for 3 h, followed by separation via SDS-PAGE and transfer onto a PVDF membrane. Ubiquitination status of PpMYB123-GST was detected using an Anti-GST antibody (TransGen Biotech, Beijing, China; dilution 1:2000).

### Statistical analyses

All experimental data were statistically analyzed using the Statistical Package for the Social Sciences (SPSS) software (IBM, Chicago, USA). The statistical significance of differences between two groups was assessed using the independent samples *t*-test, with * and ** indicating statistically significant differences at *P* < 0.05 and *P* < 0.01, respectively. Quantitative data were expressed as mean ± standard error of the mean (SEM), based on three independent biological replicates per experimental group.

## Supplementary Material

Web_Material_uhag032

## Data Availability

All data can be found online in the main text and supporting information materials.

## References

[1] Coll NS, Epple P, Dangl JL. Programmed cell death in the plant immune system. Cell Death Differ. 2011;18:1247–56 21475301 10.1038/cdd.2011.37 PMC3172094

[2] Dey N, Roy UK, Aditya M. et al. Defensive strategies of ROS in programmed cell death associated with hypertensive response in plant pathogenesis. Ann Syst Biol. 2020;3:001–9

[3] Liu Y, Zhang H. Reactive oxygen species and nitric oxide as mediators in plant hypersensitive response and stomatal closure. Plant Signal Behav. 2021;16:1985860 34668846 10.1080/15592324.2021.1985860 PMC9208772

[4] Nanda AK, Andrio E, Marino D. et al. Reactive oxygen species during plant–microorganism early interactions. J Integr Plant Biol. 2010;52:195–204 20377681 10.1111/j.1744-7909.2010.00933.x

[5] Torres MA, Jones JDG, Dangl JL. Reactive oxygen species signaling in response to pathogens. Plant Physiol. 2006;141:373–8 16760490 10.1104/pp.106.079467 PMC1475467

[6] Van Breusegem F, Dat JF. Reactive oxygen species in plant cell death. Plant Physiol. 2006;141:384–90 16760492 10.1104/pp.106.078295 PMC1475453

[7] Xu X, Chen Y, Li B. et al. Molecular mechanisms underlying multi-level defense responses of horticultural crops to fungal pathogens. Hortic Res. 2022;9:uhac066 35591926 10.1093/hr/uhac066 PMC9113409

[8] Zurbriggen MD, Carrillo N, Hajirezaei MR. ROS signaling in the hypersensitive response: when, where and what for? Plant Signal Behav. 2010;5:393–6 20383072 10.4161/psb.5.4.10793 PMC2958590

[9] Eichmann R, Richards L, Schäfer P. Hormones as go-betweens in plant microbiome assembly. Plant J. 2021;105:518–41 33332645 10.1111/tpj.15135 PMC8629125

[10] Kumar S, Korra T, Thakur R. et al. Role of plant secondary metabolites in defence and transcriptional regulation in response to biotic stress. Plant Stress. 2023;8:100154

[11] Mishra S, Roychowdhury R, Ray S. et al. Salicylic acid (SA)-mediated plant immunity against biotic stresses: an insight on molecular components and signaling mechanism. Plant Stress. 2024;11:100427

[12] Ngou BPM, Ding P, Jones JDG. Thirty years of resistance: zig-zag through the plant immune system. Plant Cell. 2022;34:1447–78 35167697 10.1093/plcell/koac041 PMC9048904

[13] Upadhyay R, Saini R, Shukla PK. et al. Role of secondary metabolites in plant defense mechanisms: a molecular and biotechnological insights. Phytochem Rev. 2025;24:953–83

[14] Cao Y, Yan X, Ran S. et al. Knockout of the lignin pathway gene *BnF5H* decreases the S/G lignin compositional ratio and improves *Sclerotinia sclerotiorum* resistance in *Brassica napus*. Plant Cell Environ. 2022;45:248–61 34697825 10.1111/pce.14208 PMC9084453

[15] Xiao S, Hu Q, Shen J. et al. GhMYB4 downregulates lignin biosynthesis and enhances cotton resistance to *Verticillium dahliae*. Plant Cell Rep. 2021;40:735–51 33638657 10.1007/s00299-021-02672-x

[16] Yang Y, He Y, Lv S. et al. The PcMYB44-mediated miR397-*PcLACs* module regulates defence-induced lignification in pear resistance to fungal disease. Mol Plant Pathol. 2023;24:1107–25 37312259 10.1111/mpp.13357 PMC10423334

[17] Daryanavard H, Postiglione AE, Mühlemann JK. et al. Flavonols modulate plant development, signaling, and stress responses. Curr Opin Plant Biol. 2023;72:102350 36870100 10.1016/j.pbi.2023.102350 PMC10372886

[18] Li T, Wang S, Shi D. et al. Phosphate deficiency induced by infection promotes synthesis of anthracnose-resistant anthocyanin-3-*O*-galactoside phytoalexins in the *Camellia sinensis* plant. Hortic Res. 2023;10:uhad222 38077497 10.1093/hr/uhad222 PMC10709544

[19] Xiao J, He M, Chen P. et al. Proanthocyanidins delay the senescence of young asparagus stems by regulating antioxidant capacity and synthesis of phytochemicals. Food Chem X. 2024;21:101222 38389577 10.1016/j.fochx.2024.101222 PMC10881539

[20] Yu D, Wei W, Fan Z. et al. VabHLH137 promotes proanthocyanidin and anthocyanin biosynthesis and enhances resistance to *Colletotrichum gloeosporioides* in grapevine. Hortic Res. 2023;10:uhac261 36778186 10.1093/hr/uhac261 PMC9907051

[21] Zhou LJ, Geng Z, Wang Y. et al. A novel transcription factor CmMYB012 inhibits flavone and anthocyanin biosynthesis in response to high temperatures in chrysanthemum. Hortic Res. 2021;8:248 34848687 10.1038/s41438-021-00675-z PMC8633327

[22] Tan S, Chen S, Zhang H. et al. The PopbZIP2–PopMYB4 regulatory module enhances disease resistance in poplars by modulating proanthocyanidin accumulation. New Phytol. 2025;246:218–36 39945234 10.1111/nph.20408

[23] Wang L, Ran L, Hou Y. et al. The transcription factor MYB115 contributes to the regulation of proanthocyanidin biosynthesis and enhances fungal resistance in poplar. New Phytol. 2017;215:351–67 28444797 10.1111/nph.14569

[24] Wang Y, Wang X, Fang J. et al. VqWRKY56 interacts with VqbZIPC22 in grapevine to promote proanthocyanidin biosynthesis and increase resistance to powdery mildew. New Phytol. 2023;237:1856–75 36527243 10.1111/nph.18688

[25] Zhao T, Huang C, Li N. et al. Ubiquitin ligase VvPUB26 in grapevine promotes proanthocyanidin synthesis and resistance to powdery mildew. Plant Physiol. 2024;195:2891–910 38688011 10.1093/plphys/kiae249

[26] Pang Y, Peel GJ, Wright E. et al. Early steps in proanthocyanidin biosynthesis in the model legume *Medicago truncatula*. Plant Physiol. 2007;145:601–15 17885080 10.1104/pp.107.107326 PMC2048810

[27] An JP, Li R, Qu FJ. et al. R2R3-MYB transcription factor MdMYB23 is involved in the cold tolerance and proanthocyanidin accumulation in apple. Plant J. 2018;96:562–77 30054966 10.1111/tpj.14050

[28] Li C, Pei J, Yan X. et al. A poplar B-box protein PtrBBX23 modulates the accumulation of anthocyanins and proanthocyanidins in response to high light. Plant Cell Environ. 2021;44:3015–33 34114251 10.1111/pce.14127

[29] Li D, Yang J, Pak S. et al. PuC3H35 confers drought tolerance by enhancing lignin and proanthocyanidin biosynthesis in the roots of *Populus ussuriensis*. New Phytol. 2022;233:390–408 34643281 10.1111/nph.17799

[30] An JP, Xu RR, Liu X. et al. Jasmonate induces biosynthesis of anthocyanin and proanthocyanidin in apple by mediating the JAZ1–TRB1–MYB9 complex. Plant J. 2021;106:1414–30 33759251 10.1111/tpj.15245

[31] An XH, Tian Y, Chen KQ. et al. *MdMYB9* and *MdMYB11* are involved in the regulation of the JA-induced biosynthesis of anthocyanin and proanthocyanidin in apples. Plant Cell Physiol. 2015;56:650–62 25527830 10.1093/pcp/pcu205

[32] Liang C, Yang B, Wei Y. et al. SA incubation induced accumulation of flavan-3-ols through activated *VvANR* expression in grape leaves. Sci Hortic. 2021;287:110269

[33] Nesi N, Jond C, Debeaujon I. et al. The Arabidopsis *TT2* gene encodes an R2R3 MYB domain protein that acts as a key determinant for proanthocyanidin accumulation in developing seed. Plant Cell. 2001;13:2099–114 11549766 10.1105/TPC.010098 PMC139454

[34] Bogs J, Jaffé FW, Takos AM. et al. The grapevine transcription factor VvMYBPA1 regulates proanthocyanidin synthesis during fruit development. Plant Physiol. 2007;143:1347–61 17208963 10.1104/pp.106.093203 PMC1820911

[35] Deluc L, Bogs J, Walker AR. et al. The transcription factor VvMYB5b contributes to the regulation of anthocyanin and proanthocyanidin biosynthesis in developing grape berries. Plant Physiol. 2008;147:2041–53 18539781 10.1104/pp.108.118919 PMC2492604

[36] Terrier N, Torregrosa L, Ageorges A. et al. Ectopic expression of VvMybPA2 promotes proanthocyanidin biosynthesis in grapevine and suggests additional targets in the pathway. Plant Physiol. 2009;149:1028–41 19098092 10.1104/pp.108.131862 PMC2633825

[37] Jiang L, Yue M, Liu Y. et al. A novel R2R3-MYB transcription factor FaMYB5 positively regulates anthocyanin and proanthocyanidin biosynthesis in cultivated strawberries (*Fragaria* × *ananassa*). Plant Biotechnol J. 2023;21:1140–58 36752420 10.1111/pbi.14024 PMC10214752

[38] Schaart JG, Dubos C, Romero De La Fuente I. et al. Identification and characterization of MYB–bHLH–WD40 regulatory complexes controlling proanthocyanidin biosynthesis in strawberry (*Fragaria*× *ananassa*) fruits. New Phytol. 2013;197:454–67 23157553 10.1111/nph.12017

[39] Zhao L, Sun J, Cai Y. et al. *PpHYH* is responsible for light-induced anthocyanin accumulation in fruit peel of *Prunus persica*. Tree Physiol. 2022;42:1662–77 35220436 10.1093/treephys/tpac025 PMC9366866

[40] Zhao L, Zhang Y, Sun J. et al. *PpHY5* is involved in anthocyanin coloration in the peach flesh surrounding the stone. Plant J. 2023;114:951–64 36919360 10.1111/tpj.16189

[41] Zhao L, Liu Y, Chen X. et al. Visible light induces the *PpHYH* transcription to promote anthocyanin pigmentation in peach peel. Fruit Res. 2023;3:25

[42] Yan J, Cai Z, Shen Z. et al. Accumulation of proanthocyanidin monomers in two genotypes of blood-flesh peach. J Hortic Sci Biotechnol. 2017;92:513–20

[43] Zhou H, Lin-Wang K, Liao L. et al. Peach MYB7 activates transcription of the proanthocyanidin pathway gene encoding leucoanthocyanidin reductase, but not anthocyanidin reductase. Front Plant Sci. 2015;6:908 26579158 10.3389/fpls.2015.00908 PMC4620396

[44] Bi G, Hu M, Fu L. et al. The cytosolic thiol peroxidase PRXIIB is an intracellular sensor for H_2_O_2_ that regulates plant immunity through a redox relay. Nat Plants. 2022;8:1160–75 36241731 10.1038/s41477-022-01252-5

[45] Tian S, Liu C, Luo F. et al. Integrated transcriptome and metabolome reveal that SlSYTA modulates ROS responses driving resistance defense in *Solanum lycopersicum*. Hortic Res. 2024;11:uhae176 39108586 10.1093/hr/uhae176 PMC11301315

[46] Wang Y, Tan J, Wu Z. et al. STAYGREEN, STAY HEALTHY: a loss-of-susceptibility mutation in the *STAYGREEN* gene provides durable, broad-spectrum disease resistances for over 50 years of US cucumber production. New Phytol. 2019;221:415–30 30022503 10.1111/nph.15353

[47] Wu J, Yang R, Yang Z. et al. ROS accumulation and antiviral defence control by microRNA528 in rice. Nat Plants. 2017;3:1–7 10.1038/nplants.2016.203 28059073

[48] Ravaglia D, Espley RV, Henry-Kirk RA. et al. Transcriptional regulation of flavonoid biosynthesis in nectarine (*Prunus persica*) by a set of R2R3 MYB transcription factors. BMC Plant Biol. 2013;13:1–14 23617716 10.1186/1471-2229-13-68 PMC3648406

[49] Zhou H, Peng Q, Zhao J. et al. Multiple R2R3-MYB transcription factors involved in the regulation of anthocyanin accumulation in peach flower. Front Plant Sci. 2016;7:1557 27818667 10.3389/fpls.2016.01557 PMC5073212

[50] Ling Q, Broad W, Trösch R. et al. Ubiquitin-dependent chloroplast-associated protein degradation in plants. Science. 2019;363:eaav4467 30792274 10.1126/science.aav4467

[51] Liu X, Zhou Y, Chen K. et al. Phosphorylation status of CPK28 affects its ubiquitination and protein stability. New Phytol. 2023;237:1270–84 36333900 10.1111/nph.18596

[52] Potel CM, Kurzawa N, Becher I. et al. Impact of phosphorylation on thermal stability of proteins. Nat Methods. 2021;18:757–9 34140700 10.1038/s41592-021-01177-5

[53] Trujillo M, Shirasu K. Ubiquitination in plant immunity. Curr Opin Plant Biol. 2010;13:402–8 20471305 10.1016/j.pbi.2010.04.002

[54] Cho SK, Ryu MY, Song C. et al. *Arabidopsis* PUB22 and PUB23 are homologous U-Box E3 ubiquitin ligases that play combinatory roles in response to drought stress. Plant Cell. 2008;20:1899–914 18664614 10.1105/tpc.108.060699 PMC2518226

[55] Jiang MG, Yang YY, Wei W. et al. Interaction of MaERF11 with the E3 ubiquitin ligase MaRFA1 is involved in the regulation of banana starch degradation during postharvest ripening. Hortic Plant J. 2025;11:608–18

[56] Park JJ, Yi J, Yoon J. et al. *OsPUB15*, an E3 ubiquitin ligase, functions to reduce cellular oxidative stress during seedling establishment. Plant J. 2011;65:194–205 21223385 10.1111/j.1365-313X.2010.04416.x

[57] Sharma M, Pandey A, Pandey GK. Role of plant U-BOX (PUB) protein in stress and development. Plant Stress. 2013;7:1–9

[58] Gaona MR, Van Tuinen A, Schipper D. et al. Mutation of *PUB17* in tomato leads to reduced susceptibility to necrotrophic fungi. Plant Biotechnol J. 2023;21:2157–9 37735839 10.1111/pbi.14127 PMC10579703

[59] Mou B, Zhao G, Wang J. et al. The OsCPK17–OsPUB12–OsRLCK176 module regulates immune homeostasis in rice. Plant Cell. 2024;36:987–1006 37831412 10.1093/plcell/koad265 PMC10980343

[60] Zhao P, Yang H, Sun Y. et al. Targeted MYC2 stabilization confers citrus Huanglongbing resistance. Science. 2025;388:191–8 40208996 10.1126/science.adq7203

[61] Han Z, Xiong D, Schneiter R. et al. The function of plant PR1 and other members of the CAP protein superfamily in plant-pathogen interactions. Mol Plant Pathol. 2023;24:651–68 36932700 10.1111/mpp.13320 PMC10189770

[62] Liu C, Liu Q, Mou Z. Redox signaling and oxidative stress in systemic acquired resistance. J Exp Bot. 2024;75:4535–48 38693779 10.1093/jxb/erae193

[63] Vlot AC, Sales JH, Lenk M. et al. Systemic propagation of immunity in plants. New Phytol. 2021;229:1234–50 32978988 10.1111/nph.16953

[64] Zeng HY, Wu YL, Xu LB. et al. Banana defense response against pathogens: breeding disease-resistant cultivars. Hortic Plant J. 2026;12:62–72

[65] Luo M, Meng FZ, Tan Q. et al. Identification, genetic diversity, and chemical control of *Xanthomonas arboricola* pv. *pruni* in China. Plant Dis. 2022;106:2415–23 35171643 10.1094/PDIS-09-21-2048-RE

[66] Socquet-Juglard D, Patocchi A, Pothier JF. et al. Evaluation of *Xanthomonas arboricola* pv. *pruni* inoculation techniques to screen for bacterial spot resistance in peach and apricot. J Plant Pathol. 2012;94:91–6

[67] Zhou H, Lin-Wang K, Wang F. et al. Activator-type R2R3-MYB genes induce a repressor-type R2R3-MYB gene to balance anthocyanin and proanthocyanidin accumulation. New Phytol. 2019;221:1919–34 30222199 10.1111/nph.15486

[68] Initiative IPG, Verde I, Abbott AG. et al. The high-quality draft genome of peach (*Prunus persica*) identifies unique patterns of genetic diversity, domestication and genome evolution. Nat Genet. 2013;45:487–94 23525075 10.1038/ng.2586

[69] Wang K, Gao Y, Peng X. et al. Using FAM labeled DNA oligos to do RNA electrophoretic mobility shift assay. Mol Biol Rep. 2010;37:2871–5 19784797 10.1007/s11033-009-9841-7

[70] An JP, Zhao L, Cao YP. et al. The SMXL8-AGL9 module mediates crosstalk between strigolactone and gibberellin to regulate strigolactone-induced anthocyanin biosynthesis in apple. Plant Cell. 2024;36:4404–25 38917246 10.1093/plcell/koae191 PMC11448916

