# Supplementary material for: PpMYB123-mediated proanthocyanidin accumulation alleviates bacterial spot disease in peach
Source: Hortic Res. 2026 Jan 30;13(5):uhag032. doi: 10.1093/hr/uhag032 (PMC13148162; doi:10.1093/hr/uhag032)
Supplement: Web_Material_uhag032 [file web_material_uhag032.zip › 3.SupplementaryA file-Revised.docx]

Table. S1 Primers for qRT-PCR analyses in peach

| **Name** | **Forward (5’→3’)** | **Reverse (5’→3’)** |
| --- | --- | --- |
| PpLAR | CTACGGTGATGGCAGCGTTA | TCTGGTATGCGGTTCTCTGC |
| PpANR | TCTCATCACAGTCATCCCTTCTC | CAAGGCATGATTTATGAGGAAGT |
| PpMYB123 | actggaacaccactttgggg | tgaagcattggccgtctgat |
| PpMYBPA1 | tggtctctcatcgccggta | accctagtgggttttgggag |
| PpPUB23 | ggggagtttatcgacgcctt | ccaagggcagagcttgatga |
| PpActin | TGCCATTGAAATCCTGAAAC | ACCAATTGGATCATCCTCCT |
| NtANR1 | CATTTGACTTTCCCAAACGC | ATTGGGCTTTTGAGTTGTGC |
| NtANR2 | TGTTCCCACTTGGGATGATA | TGCACCTATACTCTGTTAGTGGC |
| NtLAR | TCAAGGTCCTTTACGCCATC | ACGAACCTGCTTCTCTTTGG |
| Ntactin | AATGGAACTGGAATGGTCAAGGC | CCAGATCTTCTCCATGTCATCCCA |
| AtANR | AGCTCGTGCCCATTTGTTTC | CGCAATCTCTGGAACACTTGTG |
| Atactin | GCTGAGAGATTCAGATGCCCA | GTGGATTCCAGCAGCTTCCAT |
|  |  |  |

Table. S2 Primers for vector construction

| **Primer name** | | **Direction (5’→3’)** | | **Enzyme site** | |
| --- | --- | --- | --- | --- | --- |
| pSAK-PpMYB123-F | | ctagtggatccaaagaattc atggggagaagtccatgttg | | EcoRI | |
| pSAK-PpMYB123-R | | tcgagaagctttttgaattc ttaggaaatattatcttgcg | | EcoRI | |
| pSAK-PpPUB23-F | | ctagtggatccaaagaattc ATGGATCAAGAAATTGATGT | | EcoRI | |
| pSAK-PpPUB23-R | | tcgagaagctttttgaattc CTATGAAGGATAAGAAGAAA | | EcoRI | |
| PpMYB123-T1-F | | gtgagtaaggttaccgaattc TGAAGCATTGGCCGTCTGAT | | EcoRI | |
| PpMYB123-T1-R | | cgtgagctcggtaccggatcc ACTGGAACACCACTTTGGGG | | BamHI | |
| PpMYB123-T2-F | | gtgagtaaggttaccgaattc AACCAAGGCCACAAGGTTGA | | EcoRI | |
| PpMYB123-T2-R | | cgtgagctcggtaccggatcc TGGCAGAGACTGAAACGCAT | | BamHI | |
| AD-PpMYB123-F | | gccatggaggccagtgaattc atggggagaagtccatgttg | | EcoRI | |
| AD-PpMYB123-R | | cagctcgagctcgatggatcc ttaggaaatattatcttgcg | | BamHI | |
| PpPUB23-T1-F | | gtgagtaaggttaccgaattc CCAAGGGCAGAGCTTGATGA | | EcoRI | |
| PpPUB23-T1-R | | cgtgagctcggtaccggatcc ATCTCAGCGAAGACGCACTC | | BamHI | |
| PpPUB23-T2-F | | gtgagtaaggttaccgaattc GGGATAACCTACGACCGGGA | | EcoRI | |
| PpPUB23-T2-R | | cgtgagctcggtaccggatcc CTGGGTTTTGTTGACAGGCG | | BamHI | |
| pAbAi-proANR-F | | AAGCTTGAATTCGAGCTC aaaactggcaccggaaaatc | | KpnI | |
| pAbAi-proANR-R | | TACATACAGAGCACATGC CGTCTCCTCTAGAACGCGAG | | XhoI | |
| pAbAi-proLAR-F | | AAGCTTGAATTCGAGCTC caccggttccgtttgtatac | | KpnI | |
| pAbAi-proLAR-R | | TACATACAGAGCACATGC GGCTGGCTGCTGGCTGCTGG | | XhoI | |
| pGreen-proANR-F | | ctatagggcgaattgggtacc aaaactggcaccggaaaatc | | KpnI | |
| pGreen-proANR-R | | cgctctagaactagtggatcc CGTCTCCTCTAGAACGCGAG | | BamHI | |
| pGreen-proLAR-F | | ctatagggcgaattgggtacc caccggttccgtttgtatac | | KpnI | |
| pGreen-proLAR-R | | cgctctagaactagtggatcc GGCTGGCTGCTGGCTGCTGG | | BamHI | |
| BD-PpMYB123-F | | GCATATGGCCATGGAGGCC atggggagaagtccatgttg | | EcoRI | |
| BD-PpMYB123-R | | CGGCCGCTGCAGGTCGACG ttaggaaatattatcttgcg | | BamHI | |
| BD-PpPUB23-F | | GCATATGGCCATGGAGGCC atggatcaagaaattgatgt | | EcoRI | |
| BD-PpPUB23-R | | CGGCCGCTGCAGGTCGACG ctatgaaggataagaagaaa | | BamHI | |
| FAM-proANR-P1-F | | gttagctgat**ctgttg**gattgttgtt | |  | |
| FAM-proANR-P1-R | | aacaacaatc**caacag**atcagctaac | |  | |
| FAM-proANR-P2-F | | ttaagtgaaa**ccgttg**gaagacttgg | |  | |
| FAM-proANR-P2-R | | ccaagtcttc**caacgg**tttcacttaa | |  | |
| FAM-proANR-P3-F | | tttccttgat**caactg**ttcccaGAAT | |  | |
| FAM-proANR-P3-R | | ATTCtgggaa**cagttg**atcaaggaaa | |  | |
| FAM-proANR-P3-Mu-F | | tttccttgat**TTTTTT**ttcccaGAAT | |  | |
| FAM-proANR-P3-Mu-R | | ATTCtgggaa**AAAAAA**atcaaggaaa | |  | |
| FAM-proLAR-P1-F | | agccccatct**ctgttg**agctgacttg | |  | |
| FAM-proLAR-P1-R | | caagtcagct**caacag**agatggggct | |  | |
| FAM-proLAR-P2-F | | ccggatcaag**ccgttg**cgacgtgcct | |  | |
| FAM-proLAR-P2-R | | aggcacgtcg**caacgg**cttgatccgg | |  | |
| FAM-proLAR-P3-F | | ttttggaacc**caactg**ggaattgttt | |  | |
| FAM-proLAR-P3-R | | aaacaattcc**cagttgg**gttccaaaa | |  | |
| FAM-proLAR-P3-Mu-F | | ttttggaacc**TTTTTT**ggaattgttt | |  | |
| FAM-proLAR-P3-Mu-R | | aaacaattcc**AAAAAA**gttccaaaa | |  | |
| pET-PpMYB123-F | | CCATGGCTGATATCGGATCC atggggagaagtccatgttg | | EcoRI | |
| pET-PpMYB123-R | | CAAGCTTGTCGACGGAGCTC ttaggaaatattatcttgcg | | EcoRI | |
| pET-PpPUB23-F | | CCATGGCTGATATCGGATCC atggatcaagaaattgatgt | | EcoRI | |
| pET-PpPUB23-R | | CAAGCTTGTCGACGGAGCTC ctatgaaggataagaagaaa | | EcoRI | |
| pGEX-PpMYB123-F | | AATCGGATCTGGTTCCGCGT atggggagaagtccatgttg | | BamHI | |
| pGEX -PpMYB123-R | | GGCCGCTCGAGTCGACCCGG ttaggaaatattatcttgcg | | BamHI | |
| pGEX-PpPUB23-F | | AATCGGATCTGGTTCCGCGT atggatcaagaaattgatgt | | BamHI | |
| pGEX-PpPUB23-R | | GGCCGCTCGAGTCGACCCGG ctatgaaggataagaagaaa | | BamHI | |


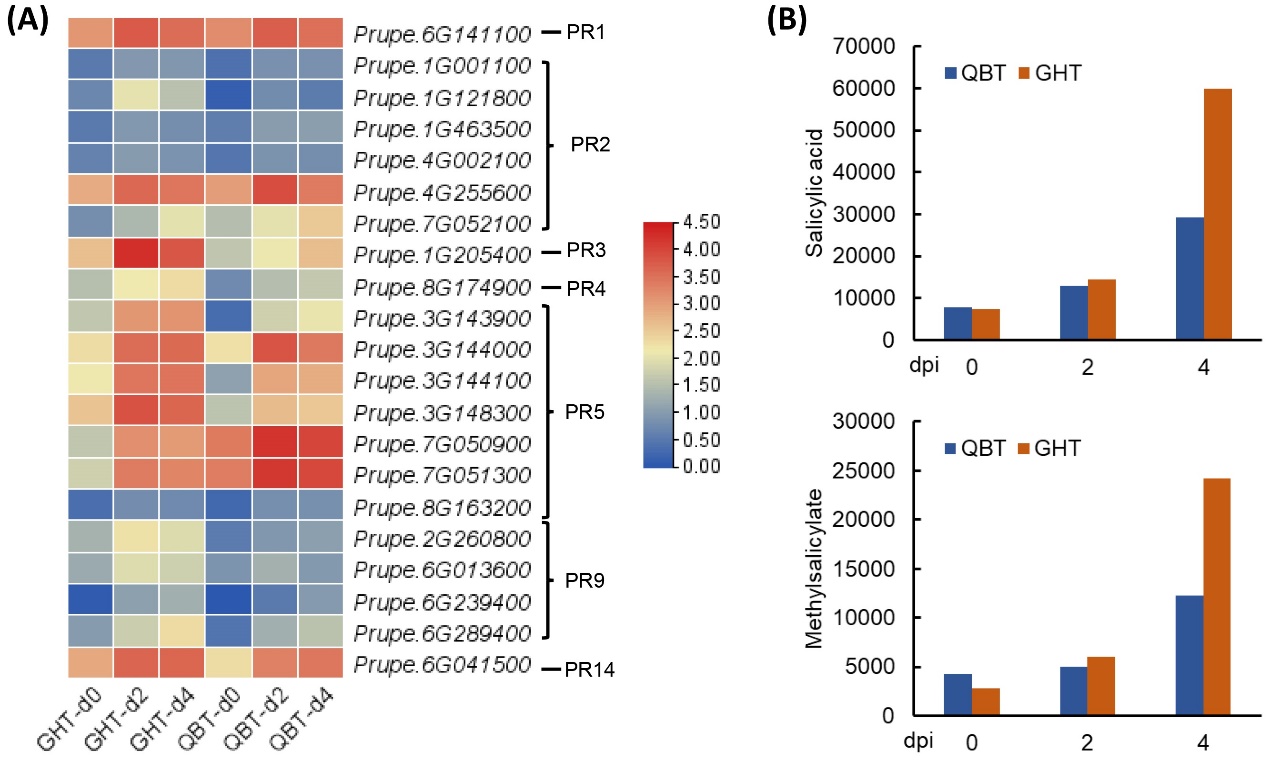


**Fig. S1.** Determination of *PR* genes expression and SA levels in leaf tissues at the site of pathogen inoculation. (A) Pathogen inoculation alters the expression profiles of *PR* genes. (B) Pathogen inoculation alters the contents of SA.


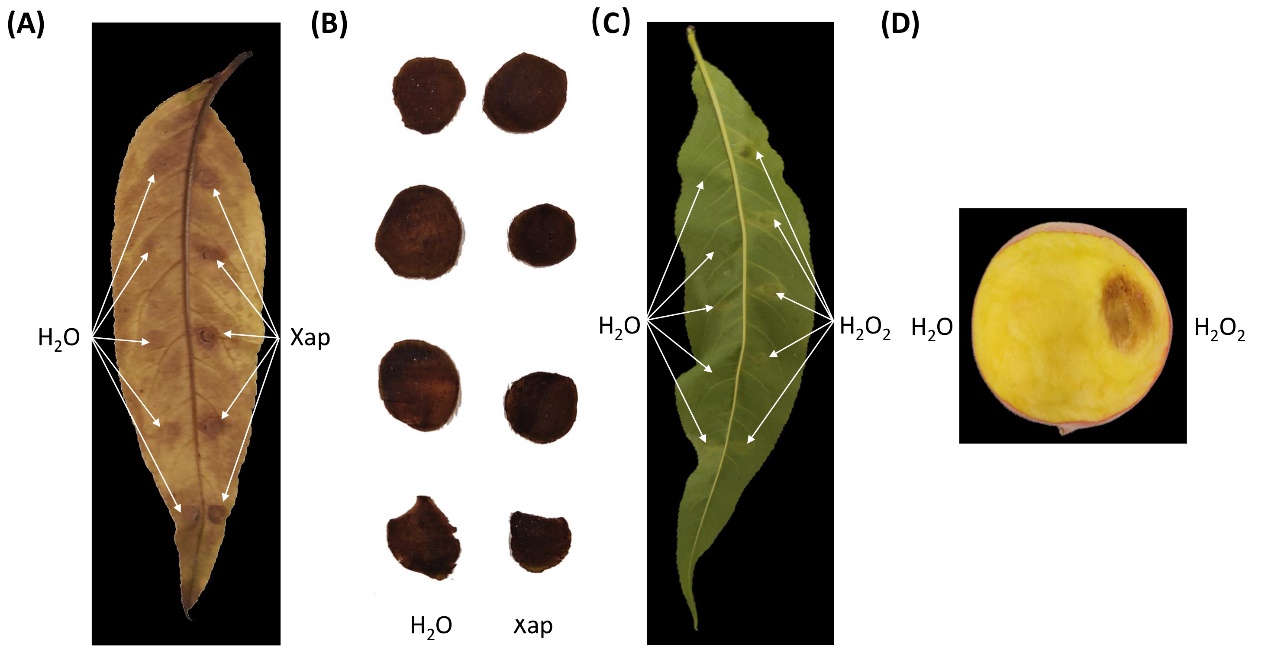


**Fig. S2.** DAB staining and effect of exogenous H_2_O_2_-treatment in peach tissues. (A) DAB staining of pathogen-inoculated peach leaves. (B) DAB staining of pathogen-inoculated peach fruits. (C) Exogenous H_2_O_2_-treatment in peach leaves. (D) Exogenous H_2_O_2_-treatment in peach fruits.


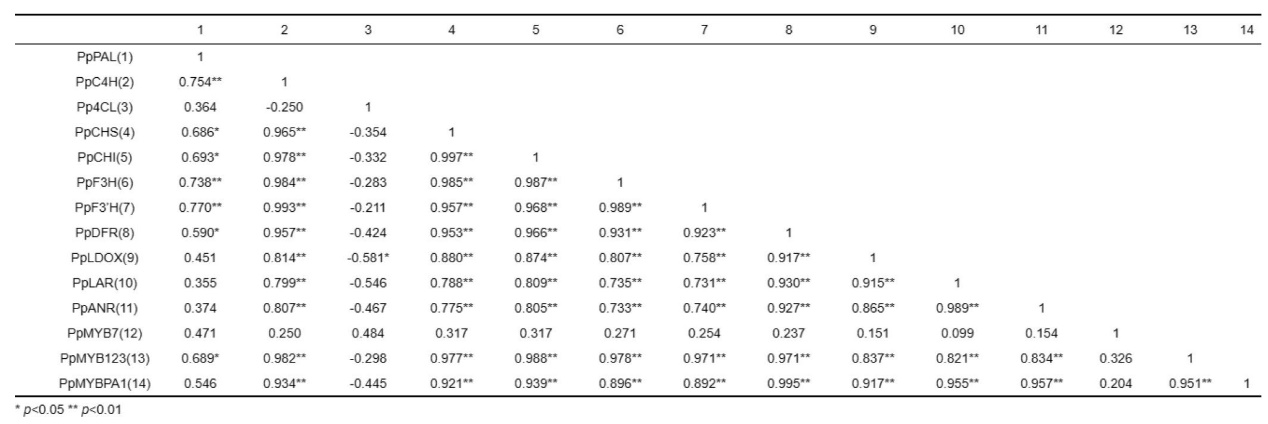


**Fig. S3.** Correlation coefficients analysis between the expression of *PpMYBs* and the expression of PA biosynthesis genes. Significance levels were determined with * representing *p* < 0.05 and ** representing *p* < 0.01.


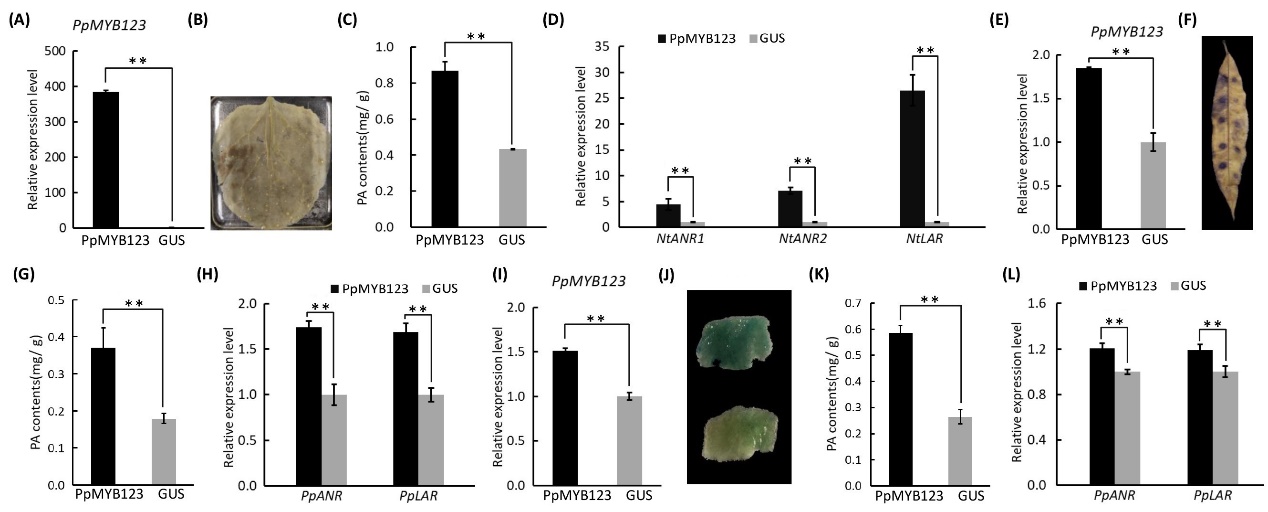


**Fig. S4.** Functional characterization of *PpMYB123* in PA biosynthesis. (A) The expression of *PpMYB123* in overexpressing and control tobacco leaves, the overexpressing of *GUS* was used as control. (B) DMACA staining of tobacco leaves. (C) Measurement of the PA content in tobacco leaves. (D) The expression profiles of *NtLAR* and *NtANR* in tobacco leaves. (E) The expression of *PpMYB123* in overexpressing and control peach leaves, the overexpressing of *GUS* was used as control. (F) DMACA staining of peach leaves. (G) Measurement of the PA content in peach leaves. (H) The expression profiles of *PpLAR* and *PptANR* in peach leaves. (I) The expression of *PpMYB123* in overexpressing and control peach fruits, the overexpressing of *GUS* was used as control. (J) DMACA staining of peach fruits. (K) Measurement of the PA content in peach fruits. (L) The expression profiles of *PpLAR* and *PptANR* in peach fruits. The Error bars show SE of three biological replicates.


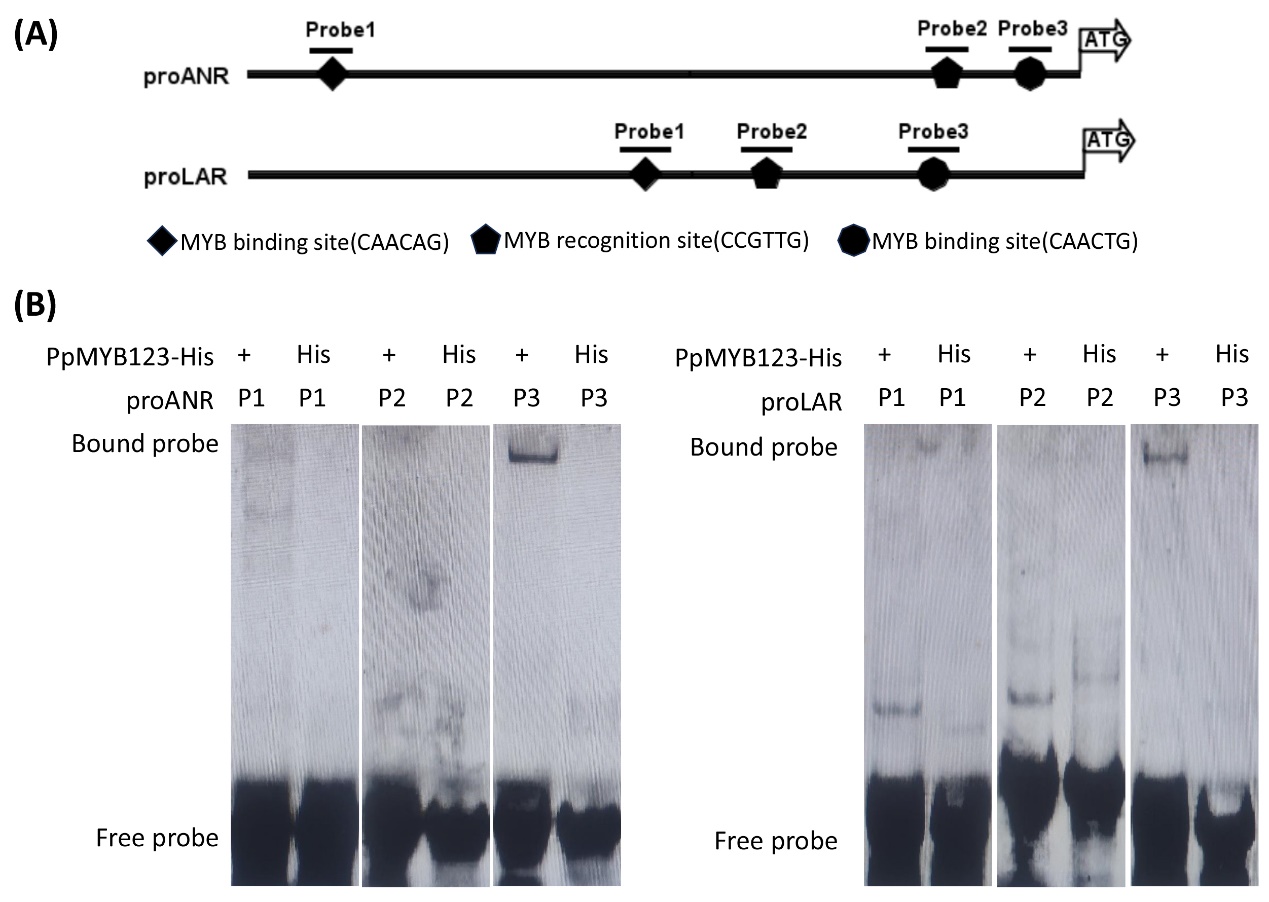


**Fig. S5.** Molecular characterization of PpMYB123 binding to *PpANR* and *PpLAR* promoter sequences. (A) Analysis of putative MYB transcription factor binding elements in *PpANR* and *PpLAR* promoters and corresponding EMSA probe design. (B) PpMYB123 specifically binds to the probe containing the MBS motif 'CAACTG' in *PpANR* and *PpLAR* promoters.


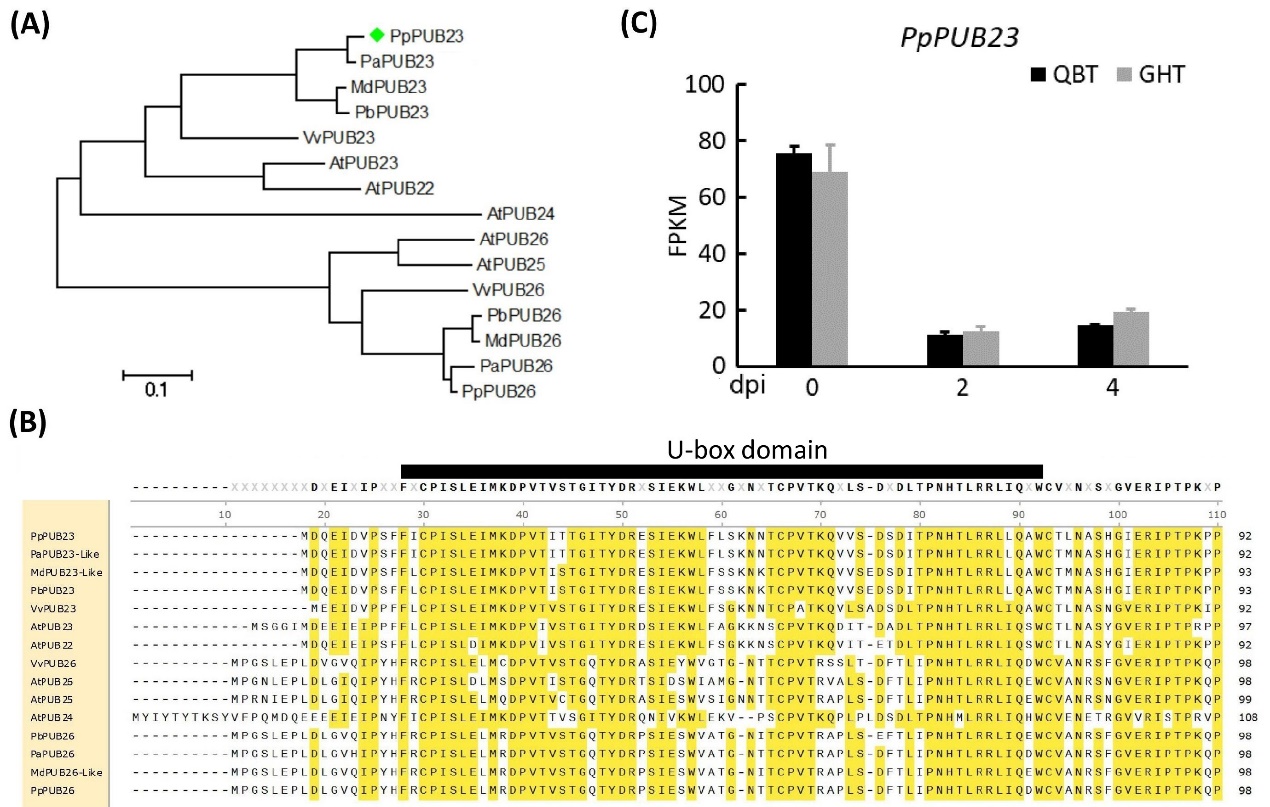


**Fig. S6.** Analysis of PpPUB23 protein sequence and expression profiles. (A) Phylogenetic analysis of PpPUB23 and its evolutionary relationships, with PpPUB23 highlighted by a green square. (B) Alignment of amino acid sequences of *PPUB23* gene in peach and its homologous genes in other plant species. (C) Quantitative analysis of PpPUB23 expression dynamics (FPKM) during pathogen inoculation in peach leaves.
