# Supplementary material for: PpMYB123-mediated proanthocyanidin accumulation alleviates bacterial spot disease in peach
Source: Hortic Res. 2026 Jan 30;13(5):uhag032. doi: 10.1093/hr/uhag032 (PMC13148162; doi:10.1093/hr/uhag032)
Supplement: Web_Material_uhag032 [file web_material_uhag032.zip › SupplementaryA Table1.docx]

Table. S1 Primers for qRT-PCR analyses in peach

| **Name** | **Forward (5’→3’)** | **Reverse (5’→3’)** |
| --- | --- | --- |
| PpLAR | CTACGGTGATGGCAGCGTTA | TCTGGTATGCGGTTCTCTGC |
| PpANR | TCTCATCACAGTCATCCCTTCTC | CAAGGCATGATTTATGAGGAAGT |
| PpMYB123 | actggaacaccactttgggg | tgaagcattggccgtctgat |
| PpMYBPA1 | tggtctctcatcgccggta | accctagtgggttttgggag |
| PpPUB23 | ggggagtttatcgacgcctt | ccaagggcagagcttgatga |
| PpActin | TGCCATTGAAATCCTGAAAC | ACCAATTGGATCATCCTCCT |
| NtANR1 | CATTTGACTTTCCCAAACGC | ATTGGGCTTTTGAGTTGTGC |
| NtANR2 | TGTTCCCACTTGGGATGATA | TGCACCTATACTCTGTTAGTGGC |
| NtLAR | TCAAGGTCCTTTACGCCATC | ACGAACCTGCTTCTCTTTGG |
| Ntactin | AATGGAACTGGAATGGTCAAGGC | CCAGATCTTCTCCATGTCATCCCA |
| AtANR | AGCTCGTGCCCATTTGTTTC | CGCAATCTCTGGAACACTTGTG |
| Atactin | GCTGAGAGATTCAGATGCCCA | GTGGATTCCAGCAGCTTCCAT |
|  |  |  |
