# Supplementary material for: PpMYB123-mediated proanthocyanidin accumulation alleviates bacterial spot disease in peach
Source: Hortic Res. 2026 Jan 30;13(5):uhag032. doi: 10.1093/hr/uhag032 (PMC13148162; doi:10.1093/hr/uhag032)
Supplement: Web_Material_uhag032 [file web_material_uhag032.zip › SupplementaryA Table2.docx]

Table. S2 Primers for vector construction

| **Primer name** | | **Direction (5’→3’)** | | **Enzyme site** | |
| --- | --- | --- | --- | --- | --- |
| pSAK-PpMYB123-F | | ctagtggatccaaagaattc atggggagaagtccatgttg | | EcoRI | |
| pSAK-PpMYB123-R | | tcgagaagctttttgaattc ttaggaaatattatcttgcg | | EcoRI | |
| pSAK-PpPUB23-F | | ctagtggatccaaagaattc ATGGATCAAGAAATTGATGT | | EcoRI | |
| pSAK-PpPUB23-R | | tcgagaagctttttgaattc CTATGAAGGATAAGAAGAAA | | EcoRI | |
| PpMYB123-T1-F | | gtgagtaaggttaccgaattc TGAAGCATTGGCCGTCTGAT | | EcoRI | |
| PpMYB123-T1-R | | cgtgagctcggtaccggatcc ACTGGAACACCACTTTGGGG | | BamHI | |
| PpMYB123-T2-F | | gtgagtaaggttaccgaattc AACCAAGGCCACAAGGTTGA | | EcoRI | |
| PpMYB123-T2-R | | cgtgagctcggtaccggatcc TGGCAGAGACTGAAACGCAT | | BamHI | |
| AD-PpMYB123-F | | gccatggaggccagtgaattc atggggagaagtccatgttg | | EcoRI | |
| AD-PpMYB123-R | | cagctcgagctcgatggatcc ttaggaaatattatcttgcg | | BamHI | |
| PpPUB23-T1-F | | gtgagtaaggttaccgaattc CCAAGGGCAGAGCTTGATGA | | EcoRI | |
| PpPUB23-T1-R | | cgtgagctcggtaccggatcc ATCTCAGCGAAGACGCACTC | | BamHI | |
| PpPUB23-T2-F | | gtgagtaaggttaccgaattc GGGATAACCTACGACCGGGA | | EcoRI | |
| PpPUB23-T2-R | | cgtgagctcggtaccggatcc CTGGGTTTTGTTGACAGGCG | | BamHI | |
| pAbAi-proANR-F | | AAGCTTGAATTCGAGCTC aaaactggcaccggaaaatc | | KpnI | |
| pAbAi-proANR-R | | TACATACAGAGCACATGC CGTCTCCTCTAGAACGCGAG | | XhoI | |
| pAbAi-proLAR-F | | AAGCTTGAATTCGAGCTC caccggttccgtttgtatac | | KpnI | |
| pAbAi-proLAR-R | | TACATACAGAGCACATGC GGCTGGCTGCTGGCTGCTGG | | XhoI | |
| pGreen-proANR-F | | ctatagggcgaattgggtacc aaaactggcaccggaaaatc | | KpnI | |
| pGreen-proANR-R | | cgctctagaactagtggatcc CGTCTCCTCTAGAACGCGAG | | BamHI | |
| pGreen-proLAR-F | | ctatagggcgaattgggtacc caccggttccgtttgtatac | | KpnI | |
| pGreen-proLAR-R | | cgctctagaactagtggatcc GGCTGGCTGCTGGCTGCTGG | | BamHI | |
| BD-PpMYB123-F | | GCATATGGCCATGGAGGCC atggggagaagtccatgttg | | EcoRI | |
| BD-PpMYB123-R | | CGGCCGCTGCAGGTCGACG ttaggaaatattatcttgcg | | BamHI | |
| BD-PpPUB23-F | | GCATATGGCCATGGAGGCC atggatcaagaaattgatgt | | EcoRI | |
| BD-PpPUB23-R | | CGGCCGCTGCAGGTCGACG ctatgaaggataagaagaaa | | BamHI | |
| FAM-proANR-P1-F | | gttagctgat**ctgttg**gattgttgtt | |  | |
| FAM-proANR-P1-R | | aacaacaatc**caacag**atcagctaac | |  | |
| FAM-proANR-P2-F | | ttaagtgaaa**ccgttg**gaagacttgg | |  | |
| FAM-proANR-P2-R | | ccaagtcttc**caacgg**tttcacttaa | |  | |
| FAM-proANR-P3-F | | tttccttgat**caactg**ttcccaGAAT | |  | |
| FAM-proANR-P3-R | | ATTCtgggaa**cagttg**atcaaggaaa | |  | |
| FAM-proANR-P3-Mu-F | | tttccttgat**TTTTTT**ttcccaGAAT | |  | |
| FAM-proANR-P3-Mu-R | | ATTCtgggaa**AAAAAA**atcaaggaaa | |  | |
| FAM-proLAR-P1-F | | agccccatct**ctgttg**agctgacttg | |  | |
| FAM-proLAR-P1-R | | caagtcagct**caacag**agatggggct | |  | |
| FAM-proLAR-P2-F | | ccggatcaag**ccgttg**cgacgtgcct | |  | |
| FAM-proLAR-P2-R | | aggcacgtcg**caacgg**cttgatccgg | |  | |
| FAM-proLAR-P3-F | | ttttggaacc**caactg**ggaattgttt | |  | |
| FAM-proLAR-P3-R | | aaacaattcc**cagttgg**gttccaaaa | |  | |
| FAM-proLAR-P3-Mu-F | | ttttggaacc**TTTTTT**ggaattgttt | |  | |
| FAM-proLAR-P3-Mu-R | | aaacaattcc**AAAAAA**gttccaaaa | |  | |
| pET-PpMYB123-F | | CCATGGCTGATATCGGATCC atggggagaagtccatgttg | | EcoRI | |
| pET-PpMYB123-R | | CAAGCTTGTCGACGGAGCTC ttaggaaatattatcttgcg | | EcoRI | |
| pGEX-PpPUB23-F | | AATCGGATCTGGTTCCGCGT atggatcaagaaattgatgt | | BamHI | |
| pGEX-PpPUB23-R | | GGCCGCTCGAGTCGACCCGG ctatgaaggataagaagaaa | | BamHI | |
